# Endothelial cell polarity and extracellular matrix composition require functional ATP6AP2 during developmental and pathological angiogenesis

**DOI:** 10.1172/jci.insight.154379

**Published:** 2022-10-10

**Authors:** Nehal R. Patel, Rajan K C, Avery Blanks, Yisu Li, Minolfa C. Prieto, Stryder M. Meadows

**Affiliations:** 1 Cell and Molecular Biology Department, Tulane University, New Orleans, Louisiana, USA.; 2 Department of Physiology, Tulane University School of Medicine, and; 3 Tulane Renal and Hypertension Center of Excellence, New Orleans, Louisiana, USA.; 4 Tulane Brain Institute, Tulane University, New Orleans, Louisiana, USA.

**Keywords:** Angiogenesis, Vascular Biology, Endothelial cells, Molecular biology, Mouse models

## Abstract

The (Pro)renin receptor ([P]RR), also known as ATP6AP2, is a single-transmembrane protein that is implicated in a multitude of biological processes. However, the exact role of ATP6AP2 during blood vessel development remains largely undefined. Here, we use an inducible endothelial cell–specific (EC-specific) *Atp6ap2*-KO mouse model to investigate the role of ATP6AP2 during both physiological and pathological angiogenesis in vivo. We observed that postnatal deletion of *Atp6ap2* in ECs results in cell migration defects, loss of tip cell polarity, and subsequent impairment of retinal angiogenesis. In vitro, *Atp6ap2*-deficient ECs similarly displayed reduced cell migration, impaired sprouting, and defective cell polarity. Transcriptional profiling of ECs isolated from *Atp6ap2* mutant mice further indicated regulatory roles in angiogenesis, cell migration, and extracellular matrix composition. Mechanistically, we provided evidence that expression of various extracellular matrix components is controlled by ATP6AP2 via the ERK pathway. Furthermore, *Atp6ap2*-deficient retinas exhibited reduced revascularization in an oxygen-induced retinopathy model. Collectively, our results demonstrate a critical role of ATP6AP2 as a regulator of developmental and pathological angiogenesis.

## Graphical abstract

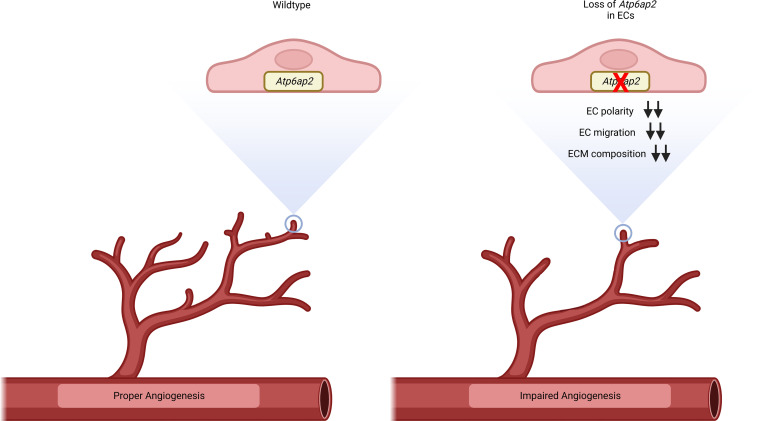

## Introduction

The growth and expansion of the vascular system is a critical physiological process occurring throughout development and adulthood, and pathological conditions can arise with dysregulation of this process. Angiogenesis is the multifaceted process whereby an initial vascular plexus is extended and elaborated throughout the body. A balance in the angiogenic growth of blood vessels is crucial for proper functioning of the vital organs, and both increases and decreases in angiogenesis have been associated with disease states. For example, ischemia secondary to impaired angiogenesis has been implicated in life-threatening disorders such as stroke, myocardial infraction, and neurodegenerative diseases (1–3). In contrast, excessive angiogenesis is linked to diseases such as diabetic retinopathy and cancer (4, 5). A better understanding of the mechanisms that regulate physiological and pathological angiogenesis will allow us to develop effective therapies that can modulate the angiogenesis process and treat vascular angiogenesis–associated disorders.

The murine retina is a well-established model system for studying angiogenesis (6, 7). During retinal angiogenesis, endothelial cells (ECs) at the vascular front rearrange into tip and stalk cells to guide vessel growth that leads to the establishment of the retinal vascular network (8). The tip ECs are proinvasive in nature and respond to guidance cues presented by the neighboring tissues and various cell types to facilitate angiogenic sprouting. Tip cell polarity is an important regulator of sprouting angiogenesis, as disruption of EC polarization has been implicated in impaired retinal angiogenesis (9–12). Furthermore, during the sprouting process, the extracellular matrix (ECM) provides a crucial scaffold for EC attachment and migration (13, 14) and modulates signal-transduction pathways essential for EC morphogenesis (15, 16). Factors that regulate retinal angiogenesis, particularly those involved with EC polarity and ECM deposition, are of great interest to the field; however, many of these critical factors remain largely uncharacterized.

The (Pro)renin receptor ([P]RR), also known as ATP6AP2, is encoded by the *Atp6ap2* gene. ATP6AP2 is ubiquitously expressed and highly conserved from mice to humans (17, 18). ATP6AP2 binds renin and prorenin as its natural ligands. The binding of ATP6AP2 to prorenin generates active renin, whereas binding to renin amplifies the hydrolysis of angiotensinogen to angiotensin I in the renin angiotensin system (RAS) cascade. These receptor-ligand interactions enhance the activity of the tissue RAS, which is implicated in various pathophysiological conditions (18–21). However, activation of ATP6AP2 by renin or prorenin also stimulates intracellular tyrosine phosphorylation pathways, independently of RAS signaling. In addition, ATP6AP2 serves as an adaptor for a number of different proteins, such as the vacuolar-ATPase (v-ATPase) and the Wnt receptor complex (22). These interactions highlight the ligand-dependent and ligand-independent multilevel regulatory properties of ATP6AP2. Accordingly, ATP6AP2 controls a variety of cell biological processes, including autophagy, cell cycle progression, cell polarity, development of kidney vasculature, differentiation of renin-expressing cells, and maintenance of podocytes (23–28). Deletion of *Atp6ap2* in embryonic stem cells failed to produce viable chimeras upon implantation into blastocysts, confirming the importance of ATP6AP2 in embryonic viability (29). Conditional *Atp6ap2* KO mice were generated to better decipher the roles of ATP6AP2 in normal development and the functioning of different organs (30). Cell-specific ablations of *Atp6ap2* performed in photoreceptor cells, murine cardiomyocytes, and smooth muscle cells have provided insight into the tissue-specific roles of ATP6AP2 (25, 30, 31). Initial studies reported that ATP6AP2 regulates angiogenic activity in proliferative diabetic retinopathy (32). Additionally, ATP6AP2 was shown to be expressed in infantile hemangioma patients and to regulate EC proliferation via the Wnt signaling pathway (33). In vitro studies utilizing human umbilical vein ECs (HUVECs) and implanted matrigel plug assays demonstrated that ATP6AP2 promotes angiogenic properties by inducing the ERK pathway independent of RAS signaling (34). However, the function of ATP6AP2 in vascular development and physiological angiogenesis in vivo has not been studied and, hence, remains largely unknown.

Here, we show that ATP6AP2 is a critical regulator of the angiogenesis process, specifically playing a role in the establishment of the ECM and tip cell polarity in the retina. Constitutive EC-specific ablation of *Atp6ap2* was embryonic lethal due to vascular defects, while inducible EC-specific deletion of *Atp6ap2* led to defective postnatal retinal angiogenesis. We observed impaired EC polarity in *Atp6ap2*-deficient retinas. Moreover, loss of *Atp6ap2* impaired EC sprouting and migration both in vivo and in cultured human ECs in vitro. Mechanistically, our data provide support that signaling of ATP6AP2 via the ERK1/2 pathway regulates downstream ECM targets upon binding of prorenin in ECs. Lastly, using an oxygen-induced retinopathy (OIR) model, we demonstrated that ATP6AP2 is also important for the revascularization process in pathological angiogenesis.

## Results

### Endothelial-specific deletion of Atp6ap2 results in impaired angiogenesis in vivo.

To gain insight into the role of ATP6AP2 in blood vessel development, we first examined its expression in various tissues by utilizing the available transcriptomic data from several EC databases. We used Vascular Endothelial Cell Trans-Omics Resource Database (VECTRDB) to analyze organ-specific, EC expression of *Atp6ap2* (35). *Atp6ap2* mRNA was detected at similar levels in P7 brain, liver, lung, kidney, and adult brain ECs, while cultured brain ECs showed the highest levels of *Atp6ap2* expression (Supplemental Figure 1A; supplemental material available online with this article; undefinedDS1). Examination of single-cell RNA-Seq (scRNA-Seq) data generated from P7 isolated brain ECs in the same database show comparable levels of *Atp6ap2* expression in subtypes of brain ECs, including capillary, venous, arterial, and tip cells (Supplemental Figure 1B). In addition, we surveyed scRNA-Seq data obtained from adult lung and brain ECs and vascular-associated cell types, such as mural cells, perivascular fibroblast-like cells, and astrocytes using the following database: http://betsholtzlab.org/VascularSingleCells/database.html (36, 37). Among these data sets, we observed various levels of *Atp6ap2* in the different subtype of lung and brain ECs, as well as their respective vascular support cells (Supplemental Figure 1, C and D). Evaluation of RNA-Seq data from isolated murine retinal ECs at different stages of postnatal development (38) revealed highest expression levels of *Atp6ap2* at P10 and P15 with retained expression up to P50 (Supplemental Figure 1E). Lastly, we performed Western blot analysis on different sources of ECs (transformed and primary murine and human ECs) and confirmed that ATP6AP2 is expressed at varying degrees in a multitude of different ECs (Supplemental Figure 1F). Overall, these analyses reveal that *Atp6ap2* is expressed in the endothelium, and among various types of ECs, thereby supporting a potential role for ATP6AP2 in regulating blood vessel growth and maintenance during development and into adulthood, respectively.

To investigate the role of ATP6AP2 during embryonic vascular development, conditional *Atp6ap2*-floxed female mice (*Atp6ap2*^fl/fl^; *Atp6ap2* resides on the X chromosome) were crossed with male mice expressing a *Cre* transgene under control of the *Tie2* promoter and enhancer (39) (Supplemental Figure 2A). Constitutive EC-specific deletion of *Atp6ap2* resulted in embryonic lethality, as no *Atp6ap2*^fl/Y^;*Tie2-*Cre male offspring were found to be born (Supplemental Figure 2B; Y refers to the Y chromosome). Conversely, *Atp6ap2*^fl/Y^, *Atp6ap2*^fl/WT^, and *Atp6ap2*^fl/WT^;*Tie2-Cre* mice were born and lived throughout adulthood. In order to identify the stages of lethality, timed mating was performed, and embryos at multiple stages were analyzed. Gross examination showed that, at E12.5, *Atp6ap2*^fl/Y^;*Tie2-*Cre embryos were under developed, often reduced in size, and perfused blood vessels were typically absent in the brains of *Atp6ap2* mutant embryos when compared with the control genotypes (Supplemental Figure 2C). However, we observed that the blood vessels in the yolk sac of *Atp6ap2* mutants appeared to be perfused similar to controls. *Atp6ap2*^fl/Y^;*Tie2-*Cre embryos examined at E13.5 and later were in the reabsorption process, indicating that lethality occurred at approximately E12.5. These data suggest that endothelial ATP6AP2 is essential for vascular development. However, lethal vascular defects were less likely to be associated with initial blood vessel formation because major dysfunctions in the vasculogenic process generally result in lethality by E10.5. Instead, the stage of embryonic lethality more closely correlated with defects in angiogenesis, which begins around E9.5 when new vessels form from the initial vascular plexus (40, 41).

To explore the role of endothelial ATP6AP2 during physiological angiogenesis, we generated inducible, EC-specific conditional KO (iECKO) mice of ATP6AP2 by combining the *Atp6ap2*-floxed and *Cdh5*(PAC)-CreER^T2^ mouse lines (30, 42). The resulting male *Atp6ap2*^fl/Y^;*Cdh5*(PAC)-CreER^T2^ and female *Atp6ap2*^fl/fl^;*Cdh5*(PAC)-CreER^T2^ mice that undergo gene deletion are hereafter referred to as *Atp6ap2*^iECKO^, while *Atp6ap2*^fl/Y^ and *Atp6ap2*^fl/fl^ mice lacking *Cdh5*(PAC)-CreER^T2^ are hereafter referred to as control mice.

**Figure 1:**
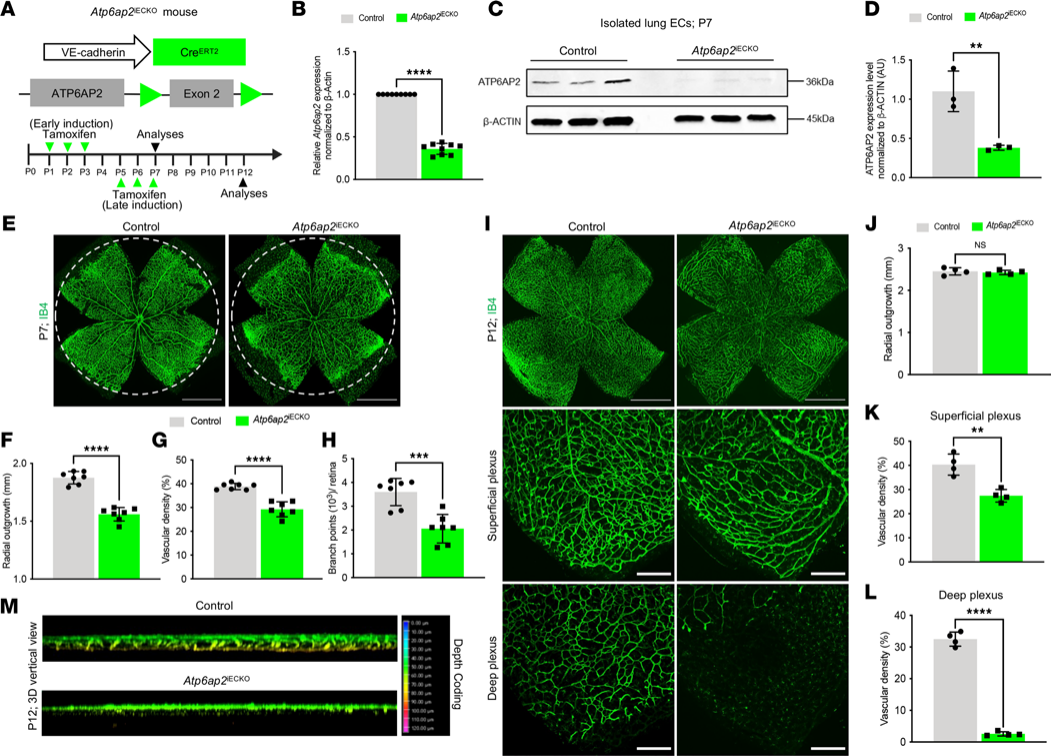
Defective retinal angiogenesis in EC-specific *Atp6ap2*-KO mice. (**A**) Strategy for EC-specific deletion of *Atp6ap2* in postnatal mice (*Atp6ap2* induced endothelial cell KO; *Atp6ap2*^iECKO^) by tamoxifen administration at P1–P3 for early induction or at P5–P7 for late induction. Murine retinas were analyzed at indicated ages. (**B**) qPCR analysis of isolated lung ECs (iLECs) show *Atp6ap2* expression levels in control and *Atp6ap2*^iECKO^ mice at P7 (*n* = 3, triplicates for each sample). (**C**) Western blot analysis of iLECs from control and *Atp6ap2*^iECKO^ mice at P7. (**D**) Densitometric quantification of ATP6AP2 levels in **C** (*n* = 3). (**E**–**H**) Whole-mount isolectin-IB4–stained (IB4-stained) retinas (dotted circles represent outgrowth in the control retina) (**E**) and quantification of radial outgrowth (**F**), vascular density (**G**), and branch points in control and *Atp6ap2*^iECKO^ mice at P7 (*n* =7) (**H**). Scale bars: 1,000 µm. (**I**–**L**) Whole-mount IB4-stained retinas and closeup images of IB4^+^ vessels in the superficial plexus and deep plexus (**I**) with quantification of radial outgrowth (*n* = 4) (**J**), vascular density in the superficial plexus (*n* = 4) (**K**), and vascular density in the deep plexus (*n* = 4) (**L**) in control and *Atp6ap2*^iECKO^ mice at P12. Scale bars: 1,000 µm. (**M**) Three-dimensional reconstructed images of the IB4^+^ deep vascular plexus and comparisons of the length of the perpendicular growth in control and *Atp6ap2*^iECKO^ mice at P12. Note very few yellow depth-coded vessels in the deep plexus of *Atp6ap2* mutants. Data are shown as mean ± SD; 2-tailed unpaired *t* test. **P < 0.01, ***P < 0.001, ****P < 0.0001.

Utilizing the *Atp6ap2*^iECKO^ and control mice, our studies centered on the angiogenic blood vessels that form in the retina during postnatal development. Cre-mediated recombination of *Atp6ap2* was induced by daily oral administration of tamoxifen at P1–P3 for early induction or at P5–P7 for late induction (Figure 1A). Successful, robust EC-specific Cre-mediated deletion of *Atp6ap2* was confirmed at the mRNA level in both isolated lung ECs (iLECs) and isolated retinal ECs (iRECs) (Figure 1B). This was also confirmed at the protein level in iLECs at P7 (Figure 1, C and D). Additionally, the Ai14 (Rosa-CAG-LSL-tdTomato) reporter line (43) indicated EC-specific Cre-mediated recombination in *Atp6ap2*^iECKO^ mice (Supplemental Figure 3). Examination of isolectin B4–stained (IB4-stained) whole-mount retinas at P7 following early induction revealed impaired retinal vascular development in *Atp6ap2*^iECKO^ mice. In comparison with control retinas, *Atp6ap2*^iECKO^ retinas exhibited a 17%, 25%, and 43% decrease in vascular outgrowth, density, and branch points, respectively (Figure 1, E–H). We also assessed for potential contributions of Cre toxicity to *Atp6ap2*^iECKO^-derived vascular phenotypes; however, no significant changes in vascular outgrowth were observed in Cre^+^ mice in the absence of gene deletion (Supplemental Figure 4). After initial peripheral outgrowth of the superficial vessel plexus, retinal vessels sprout perpendicularly into the deeper retina at approximately P7 or P8. To assess angiogenesis in the deep layer, delayed inactivation of *Atp6ap2* was performed at P5–P7, and retinas were analyzed at P12. No differences in superficial vascular outgrowth between the control and *Atp6ap2*^iECKO^ retinas were observed (Figure 1, I and J). However, P12 *Atp6ap2*^iECKO^ retinas displayed both reduced vascular density in the superficial vascular plexus (Figure 1, I and K) and severely diminished vascular sprouting into the deep layer of the retina (Figure 1, I, L, and M). Specifically, we observed a blunted vertical sprout phenotype with substantially fewer sprouts in *Atp6ap2*^iECKO^ mice in comparison with control mice that displayed proper vertical sprouting into the deep plexus (Supplemental Figure 5). Taken together, these findings indicate that ATP6AP2 is essential for retinal angiogenic growth during postnatal development.

### ATP6AP2 regulates sprouting angiogenesis.

**Figure 2:**
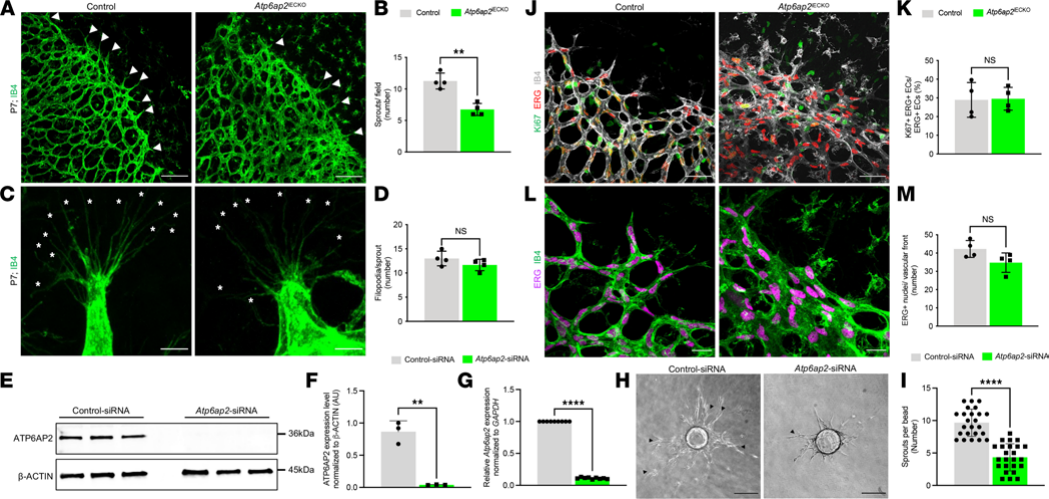
ATP6AP2 is critical for sprouting angiogenesis. (**A**) Images of IB4^+^ vessels from P7 control and *Atp6ap2*^iECKO^ retinas, with white arrowheads indicating sprouts at the vascular front. Scale bars: 100 µm. (**B**) Quantification of the number of sprouts in control and *Atp6ap2*^iECKO^ mice at P7 (*n* = 4). (**C**) Images of IB4^+^ vessels at high magnification showing filopodia in sprouts (white asterisks) at the vascular front . Scale bars: 10 µm. (**D**) Quantification of the number of filopodia per sprout in control and *Atp6ap2*^iECKO^ mice at P7 (*n* = 4). (**E**) Western blot analysis of ATP6AP2 and β-actin in control-siRNA and *Atp6ap2* siRNA–treated TeloHAECs. (**F**) Densitometric quantification of ATP6AP2 levels in **E** normalized to β-actin in TeloHAECs following siRNA treatments (*n* = 3). (**G**) qPCR analysis of control-siRNA and *Atp6ap2* siRNA–treated TeloHAECs for *Atp6ap2* mRNA levels normalized to *GAPDH* transcripts (*n* = 3; triplicates for each sample). (**H**) Representative images of TeloHAEC sprouting bead assays embedded in 3D fibrinogen gel at 120 hours following control and *Atp6ap2*-siRNA treatments. scale bars: 100 µm. Black arrowheads indicate vessel bifurcations. (**I**) Quantification of the number of sprouts per bead (*n* = 18). (**J**) Images of IB4^+^ vessels at the vascular front of control and *Atp6ap2*^iECKO^ P7 retinas immunolabeled for Ki67 and the EC-specific nuclear marker ETS transcription factor ERG. Scale bars: 50 µm. (**K**) Quantification of Ki67^+^ERG^+^ proliferative ECs in control and *Atp6ap2*^iECKO^ mice at P7 (*n* = 4). (**L**) Images of ERG^+^ EC nuclei and IB4^+^ vessels at the vascular front. Scale bars: 25 µm. (**M**) Quantification of the number of ERG^+^ ECs in control and *Atp6ap2*^iECKO^ P7 mice at the vascular front (*n* = 4). Data are shown as mean ± SD; 2-tailed unpaired *t* test. **P < 0.01, ****P < 0.0001.

The most obvious defects in *Atp6ap2*^iECKO^ retinas were observed at the vascular front, where tip cells (also known as superficial tip cells) actively guide angiogenic growth radially. To gain insight into the vascular deficiencies associated with this phenotype, we performed a detailed analysis of the retinal vascular growth front at P7. We note that the vascular density at the leading front normally varies in regions near the arteries and veins (the vasculature is generally denser near the ends of the veins), so we were careful to assess equivalent areas in our analyses. *Atp6ap2*^iECKO^ mice displayed a decrease in total number of sprouts compared with control retinas (Figure 2, A and B). Interestingly, sprouts that did form in *Atp6ap2* mutants showed no difference in filopodia numbers per sprout compared with the control group (Figure 2, C and D). To further explore the role of ATP6AP2 in sprouting angiogenesis, we analyzed sprout formation in a fibrin bead assay using immortalized human aortic ECs (telomerase human aortic EC [TeloHAECs]) because of their robust ATP6AP2 expression profile (Supplemental Figure 1F). We first treated TeloHAECs with siRNAs targeting *Atp6ap2* and verified successful siRNA-mediated downregulation at both the mRNA and protein levels compared with scrambled control siRNA–treated cells (Figure 2, E–G). Next, equal numbers of *Atp6ap2* siRNA– and control siRNA–treated TeloHAECs were coated onto beads and subjected to the fibrin bead sprouting assay. After 120 hours, *Atp6ap2-*deficient ECs displayed defective sprouting with significantly fewer sprouts per bead, and the sprouts rarely formed bifurcations when compared with the sprouts produced by control siRNA–treated cells (Figure 2, H and I, and Supplemental Figure 6). Additional DAPI staining also demonstrated that the beads were sufficiently covered by ECs in both siRNA treatments groups (Supplemental Figure 6). Based on these data, we conclude that ATP6AP2 is necessary for proper sprouting angiogenesis.

We also observed that the vasculature in *Atp6ap2* mutants was considerably dense at the growth front, with ECs more closely packed together. Compromised sprouting angiogenesis likely contributed to the increased density; however, changes in EC proliferation alone or in combination with impaired sprout formation could also explain this observed phenotype. We assessed whether proliferation in the vascular front was altered by immunofluorescence colabeling for the ETS-related protein (ERG), an EC-specific nuclear marker, and the proliferation marker Ki67. Quantification of Ki67^+^/ERG^+^ ECs showed no changes in EC proliferation at the vascular front upon loss of ATP6AP2 (Figure 2, J and K). Moreover, we found no difference in the total number of ERG^+^ ECs at the leading vessel front compared with control *Atp6ap2* retinas (Figure 2, L and M). Thus, changes in EC proliferation do not appear to be responsible for the increased vascular density at the growing front.

### Loss of ATP6AP2 leads to defective EC migration and polarity in vivo and in vitro.

**Figure 3:**
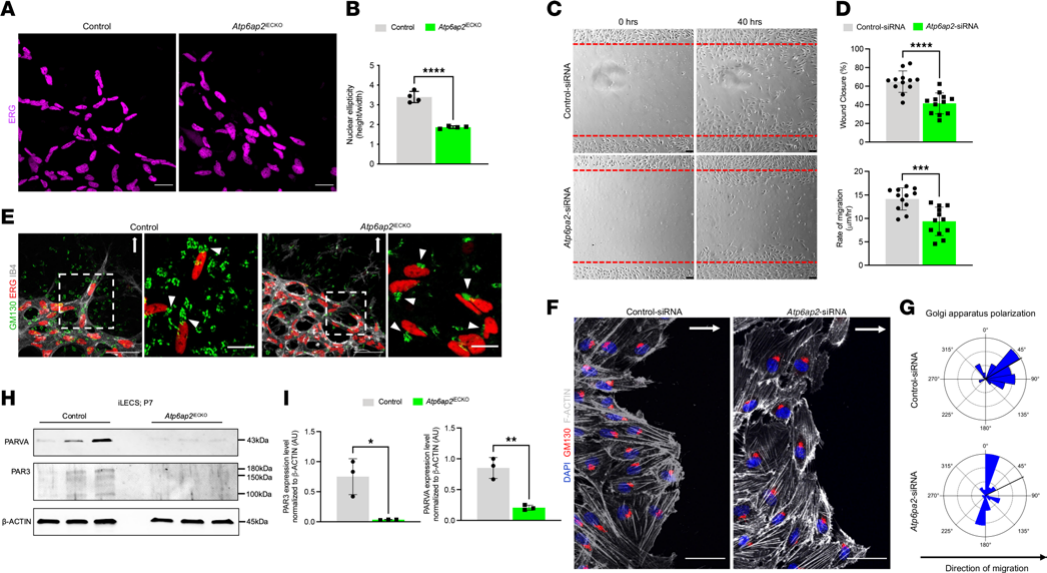
ATP6AP2 regulates endothelial cell migration and polarity in vivo and in vitro. (**A**) Representative images of ERG^+^ nuclei of ECs at the vascular front in P7 control and *Atp6ap2*^iECKO^ retinas. Scale bars: 25 µm. (**B**) Quantification of nuclear ellipticity in control and *Atp6ap2*^iECKO^ mice at P7 (*n* = 4). (**C**) Scratch wound assays performed on TeloHAEC monolayers following control and *Atp6ap2* siRNA treatments. Images at 0 and 40 hours following the scratch. Scale bars: 75 µm. (**D**) Quantification of the wound closure and rate of migration in control and *Atp6ap2* siRNA–treated TeloHAECs at 40 hours (*n* = 12). (**E**) Images of fluorescently labeled IB4^+^ vessels, ERG^+^ nuclei in ECs, and Golgi Matrix Protein GM130^+^ Golgi apparatuses at the vascular front with their respective insets (white dashed-line boxed) highlighting tip cells in control and *Atp6ap2*^iECKO^ mice at P7. Scale bars: 50 µm and 15 μm (insets). White arrowheads denote the Golgi apparatus position in respect to the nucleus. White arrows indicate direction of the retinal vasculature outgrowth. (**F**) Images of DAPI^+^ nuclei, GM130^+^ Golgi apparatuses, and F-actin^+^ cytoskeleton of indicated TeloHAECs at 12 hours after initiating cell migration in a scratch assay . Scale bars: 50 µm. White arrows indicate the direction of migration. (**G**) Rose plots showing the Golgi apparatus polarization in respect to the nucleus in both control and *Atp6ap2* siRNA–treated TeloHAECs at 12 hours after scratch (*n* = 52). (**H**) Western blot analysis of PAR3, PARVA, and β-actin from control and *Atp6ap2*^iECKO^ iLECs at P7 (*n* = 3). (**I**) Densitometric quantifications of PAR3 and PARVA levels in control and *Atp6ap2*^iECKO^ iLECs from **H**. Data are shown as mean ± SD; 2-tailed unpaired *t* test. *P < 0.05, **P < 0.01, ***P < 0.001, ****P < 0.0001.

In addition to an increased vessel density at the vascular front, *Atp6ap2*^iECKO^ retinas exhibited reduced outgrowth of the superficial vascular plexus (Figure 1, E and F). This phenotype often occurs as the result of defective EC migration, which can subsequently cause an increased vessel density at the front. To further evaluate ATP6AP2’s role in EC migration, we characterized EC nuclei shape at the vascular front by staining for ERG. We observed that the majority of the nuclei of tip ECs in the control retinas were elliptical, while the nuclei of ECs at the vascular front of *Atp6ap2*^iECKO^ mice were more spherical in shape (Figure 3, A and B). Elliptical nuclei are associated with actively migrating cells, whereas spherical nuclei are a characteristic of static cells (12, 44, 45). We next performed scratch-wound assays on siRNA-treated TeloHAECs to characterize migration in vitro. Quantification analysis of the invaded area 40 hours after scratch demonstrated that loss of *Atp6ap2* resulted in impaired wound closure and significantly reduced the rate of EC migration (Figure 3, C and D). Together, our in vivo and in vitro results further support the notion that diminished vascular sprouting and migration in the absence of ATP6AP2 contribute to reduced retinal vascular outgrowth and increased vessel density at the leading front.

Defects in directed cell migration can be caused by numerous factors. Using the scratch-wound assay, we evaluated the possible effect of cell apoptosis on wound closure rate. Immunofluorescence staining for cleaved caspase-3 (cCASP-3) was performed on both control and *Atp6ap2* siRNA–treated cells following cell migration, and essentially no cells, either at the wound site or within the culture, were positive for cCASP-3 in either group (Supplemental Figure 7, A and B). Furthermore, to expand this analysis in vivo, we performed immunofluorescence antibody staining for cCASP-3 on *Atp6ap2* control and mutant retinas. Similar to the scratch assay, we did not observe any differences in cCASP-3 staining between the samples (Supplemental Figure 7C). Together, we conclude that cell death was not associated with the loss of ATP6AP2 and, therefore, was not a contributing factor to impairment of EC migration in vitro or involved in the manifestation of the defective retinal vasculature (Supplemental Figure 7).

To further identify potential causes of defective EC migration in *Atp6ap2* mutants, we focused on assessing EC polarity. Alterations in cell polarity have substantial effects on cell migration (10, 46, 47), while ATP6AP2 has been demonstrated to regulate cell polarity dynamics in other cell types (25, 48). Typically, ECs establish a front-rear polarity axis by orienting their Golgi apparatus in front of the nucleus and toward the direction of migration (toward the retina periphery). Thus, we initially assessed tip EC polarity at the angiogenic front by staining for IB4, ERG, and Golgi matrix protein 130 (GM130) to define Golgi apparatus polarization in respect to the EC nuclei. In control retinas, tip ECs generally had their Golgi positioned in front of the nucleus toward the peripheral avascular area, while such polarization was rarely observed in the ECs at the growing front of *Atp6ap2*^iECKO^ mice (Figure 3E). Furthermore, we assessed whether the loss of ATP6AP2 affects EC polarity within arteries, veins, and capillaries. ECs in the arteries and veins typically orient the front of the cell (polarize) against the direction of the flow. Immunofluorescence staining of control and *Atp6ap2*^iECKO^ retinas at P7 for GM130, IB4, and ERG showed that loss of ATP6AP2 did not impact the polarity of ECs in the capillary plexus, veins, or the arteries (Supplemental Figure 8). Moreover, the morphology of the arteries and veins appeared indistinguishable between *Atp6ap2* control and mutant retinas. Thus, alterations in polarity appeared to be limited to the endothelial tip cells where the vascular phenotypes were most severe. Next, we investigated whether ATP6AP2 regulates endothelial polarization in vitro. siRNA-treated TeloHAECs were subjected to scratch-wound assays, and the nucleus/Golgi axis angles were measured near the wound site. The majority of control siRNA–treated cells showed polarization of GM130-stained Golgi in front of the nucleus and in the direction of the wound where migration normally occurs (Figure 3, F and G). In contrast, *Atp6ap2* siRNA–treated cells failed to consistently polarize Golgi toward the wound (Figure 3, F and G). These results were consistent with the defective tip cell polarity observed within ATP6AP2-deficient ECs in vivo and revealed a prominent role for ATP6AP2 in determining EC polarity.

Previous reports investigating photoreceptor cells have indicated a physical association between ATP6AP2 and PAR3 (25), which has been established to be a critical regulator of polarity in ECs (49, 50) and many other cell types (51, 52). Additionally, ATP6AP2 has been shown to have gene-to-gene network interactions relating to cancer cell metabolism with PARVA (53), a member of the Parvins protein family that regulates ECs polarity during embryonic blood vessel development (54). Given these relationships, we examined whether loss of ATP6AP2 had effects on the in vivo expression of either protein. Utilizing proteins from iLECs, we quantified expression levels of PAR3 and PARVA. *Atp6ap2*^iECKO^ mice displayed a substantial reduction in PAR3 and PARVA expression as compared with the control iLECs (Figure 3, H and I). Thus, these results connect ATP6AP2 deficiency to the downregulation of 2 major ECs’ polarity-determinant proteins. Collectively, our in vivo and in vitro evidence underscored the importance of ATP6AP2 in the establishment of front-rear EC polarity during angiogenic expansion of the vasculature.

### Gene expression changes in Atp6ap2-deficient ECs reveal potential roles in various cellular processes.

**Figure 4:**
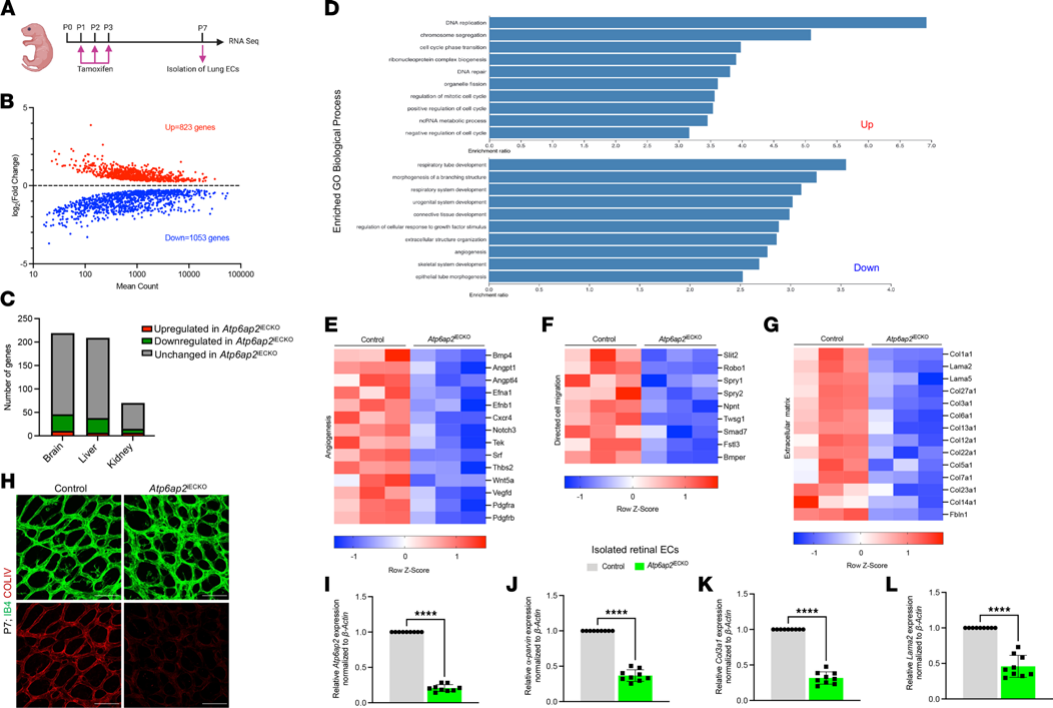
Loss of *Atp6ap2* in ECs results in misregulation of angiogenesis, directed cell migration, and extracellular matrix–related genes. (**A**) Outline of the workflow used to collect iLECs from control and *Atp6ap2*^iECKO^ mice at P7 (*n* = 3) for RNA-Seq analyses. (**B**) MA plot of differentially expressed genes between *Atp6ap2*^iECKO^ and control iLECs. Red dots, upregulated genes (823 genes); blue dots, downregulated genes (1,053 genes). (**C**) Summary of *Atp6ap2*^iECKO^-upregulated and -downregulated EC-gene distribution among organ EC–specific mRNAs. (**D**) Top GO biological process terms enriched in up- or downregulated genes in *Atp6ap2*^iECKO^ iLECs (FDR ≤ 0.05). (**E**–**G**) Representative clustered heatmaps of gene count *Z* scores for angiogenesis (**E**), cell migration (**F**), and extracellular matrix–related genes (**G**) that are differentially expressed upon loss of *Atp6ap2* in ECs. Columns represent individual biological replicates. (**H**) Images of P7 control and *Atp6ap2*^iECKO^ retinas immunofluorescently labeled for IB4 and collagen IV (COLIV). Note reduced levels of COLIV in *Atp6ap2* mutants. Scale bars: 50 μm. (**I**–**L**) qPCR analysis of isolated retinal ECs (iRECs) shows *Atp6ap2*, *α-parvin, Col3a1*, and *Lama2* expression levels in control and *Atp6ap2*^iECKO^ mice at P7 (*n* = 3, triplicates for each sample). Data are shown as mean ± SD; 2-tailed unpaired *t* test. ****P < 0.0001.

Information pertaining to the function of ATP6AP2 in the endothelium is exceedingly limited. Therefore, we performed RNA-Seq on iLECs at P7 (Figure 4A) to build an initial, comprehensive understanding of the ATP6AP2-associated processes at play in postnatal vascular development. RNA-Seq analysis of 3 biological replicates revealed 1,876 differentially expressed genes (823 upregulated genes and 1,053 downregulated genes) after *Atp6ap2* inactivation in ECs (Figure 4B). Next, we performed cross-organ comparison of the differentially expressed genes in *Atp6ap2*^iECKO^ mice with those identified as organ-specific EC transcripts (35). We observed relatively small changes in sets of organ-specific genes for the brain, liver, and kidney upon loss of ATP6AP2 (Figure 4C). Gene ontology (GO) analysis for enriched biological processes showed upregulated genes to be associated with DNA replication, regulation of cell cycle, and metabolic process (Figure 4D). In contrast, the downregulated genes were enriched in processes associated with angiogenesis, extracellular structure organization, and regulation of cellular response to growth factor stimulus (Figure 4D). Additional heatmap analyses indicate downregulation of transcripts encoded by genes involved in promoting angiogenesis (Figure 4E), cell-directed migration (Figure 4F), and ECM composition (Figure 4G) in *Atp6ap2*^iECKO^ mice compared with controls. Decreases in expression of proangiogenic and cell migration genes in *Atp6ap2* mutants further confirmed a vital role of ATP6AP2 in the regulation of angiogenesis. Lastly, GO analyses for phenotype, molecular function, cellular component, and pathway enrichment in differentially expressed genes revealed involvement of downregulated genes with abnormal blood vessel morphology, ECM structural constituent, cell leading edge, and axon guidance pathway (Supplemental Figure 9).

Based upon the retinal vascular phenotypes observed in *Atp6ap2*^iECKO^ retinas (Figures 1–3), it was not surprising that genes involved in angiogenesis and cell migration were found to be reduced in the iLECs. However, dramatic changes in expression of numerous ECM markers, primarily collagens, and laminins were somewhat unexpected. Therefore, to assess whether our ECM transcriptomic findings in the iLECs were conserved in the retina, we examined gene expression for several ECM components. Immunofluorescence analysis of collagen IV (COLIV), one of the major constituents of the basement membrane, revealed a significant reduction in expression within *Atp6ap2*^iECKO^ mice compared with controls (Figure 4H), similar to the iLEC RNA-Seq data. Fibronectin (FN), another major component of the vascular basement membrane that provides critical support for EC adhesion during vascular expansion in the retina was also evaluated by immunostaining. Comparable with the iLECs, we did not observe any obvious changes in FN expression at P7 in *Atp6ap2* mutants as compared with the control group (Supplemental Figure 10). iRECs from control and *Atp6ap2*^iECKO^ mice were used to further verify differentially expressed genes determined via RNA-Seq. Quantitative PCR (qPCR) analysis confirmed significant downregulation of *Atp6ap2* at P7 (Figure 4I), while ECM-related genes collagen III α 1 chain (*Col3a1*) and laminin subunit α 2 (*Lama2*) showed reduced expression in *Atp6ap2*^iECKO^ mice compared with controls (Figure 4, K and L). RNA-Seq data also show downregulation in mRNA expression of the cell polarity–associated gene *α-parvin* in *Atp6ap2* mutants (log_2_fold change = –0.27). PARVA is the gene product of *α-parvin*, which we previously found to be decreased in iLECs (Figure 3, H and I). Likewise, we found that the cell polarity–associated gene *α-parvin* was also substantially decreased in *Atp6ap2* mutant iRECs (Figure 4J), which reinforces our data implicating ATP6AP2’s involvement in EC polarity. Collectively, the gene expression findings in both iLECs and retinal ECs supported our previous data and further indicated that ATP6AP2 plays a critical role in multiple processes associated with angiogenesis, including those related to the ECM.

### ATP6AP2 silencing impairs prorenin-ERK–mediated pathway and downstream ECM composition in ECs.

**Figure 5:**
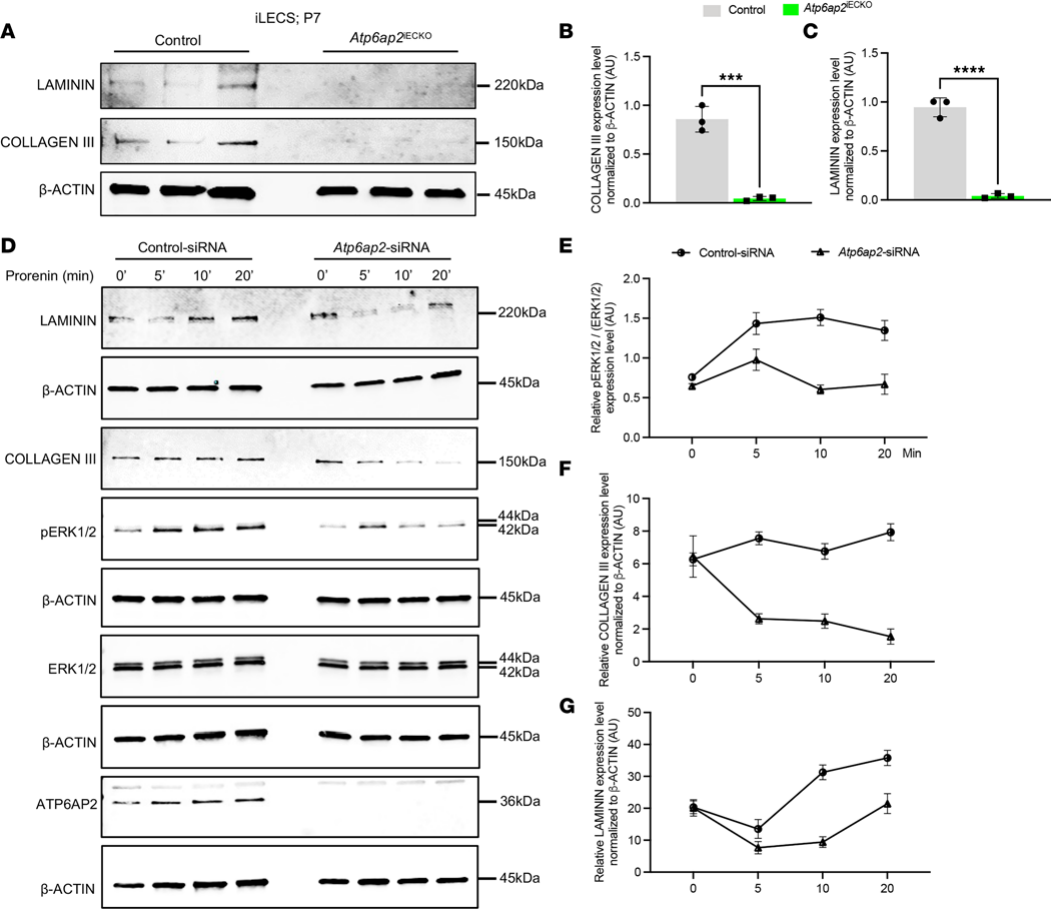
ATP6AP2 knockdown in ECs results in impaired ERK1/2 signaling and downregulation of extracellular matrix proteins. (**A**) Western blot analysis of collagen III, laminin, and β-actin from control and *Atp6ap2*^iECKO^ iLECs at P7 (*n* = 3). (**B** and **C**) Densitometric quantification of collagen III (**B**) and laminin (**C**) levels in control and *Atp6ap2*^iECKO^ iLECs from **A**. (**D**) Western blot analyses for the indicated proteins in prorenin-stimulated TeloHAECs at indicated time points following control and *Atp6ap2* siRNA treatment. Note that the β-actin blots correspond to the same protein samples represented directly above them. For instance, the β-actin blot at the bottom corresponds to the samples used to assess ATP6AP2. (**E**–**G**) Densitometric quantifications of pERK1/2 (**E**), collagen III (**F**), laminin (**G**) levels in control and *Atp6ap2* siRNA–treated cells at indicated time points following prorenin stimulation (*n* = 3 for each time point). Data are shown as mean ± SD; 2-tailed unpaired *t* test. ***P < 0.001, ****P < 0.0001.

A functional ECM is crucial to allow for proper EC adhesion and migration during expansion of the vascular network (55–57). Our RNA-Seq data and analyses of retinal ECs had revealed that ECM-related genes, especially collagens and laminins, were significantly affected by the loss of ATP6AP2 (Figure 4, G, K, and L). Additionally, we found decreased expression of COLIII and laminin in iLECs of P7 *Atp6ap2*^iECKO^ mice compared with the controls (Figure 5, A–C), further validating the RNA-Seq results and corroborating the ECM findings in the retinal vasculature. These data demonstrate that endothelial loss of ATP6AP2 leads to a marked reduction in ECM of the endothelium.

Prorenin, an inactive precursor of renin, has been shown to bind ATP6AP2 in ECs and subsequently activate the ERK1/2 pathway independently of the renin-angiotensin system (34). However, activation of this pathway and its effect on the downstream targets in ECs remain unclear. Interestingly, prorenin-ATP6AP2 activation of the ERK pathway has been shown to regulate expression of matrix factors in mesangial cells (58). These findings, coupled with our own results, led us to hypothesize that downregulation of ECM-related components observed in ATP6AP2-deficient ECs is due to impairment in the prorenin-mediated activation of ERK1/2 in ECs. To address this hypothesis, we stimulated *Atp6ap2* siRNA–treated TeloHAECs with prorenin. Following prorenin stimulation, there were no differences in expression levels of total ERK1/2 in control and *Atp6ap2* siRNA–treated cells (Figure 5D). However, expression levels for phosphorylated ERK1/2 (pERK1/2) were significantly decreased after 5, 10, and 20 minutes of prorenin stimulation in *Atp6ap2* siRNA–treated cells compared with the controls (Figure 5, D and E) and as previously reported (34). Additionally, we examined levels of COLIII and laminin following prorenin stimulation. We observed a consistent reduction of both ECM proteins in *Atp6ap2* siRNA–treated cells compared with the control siRNA–treated cells (Figure 5, D, F, and G). Based upon these findings, our data point to a role for ATP6AP2 in modulating ECM composition.

### Loss of Atp6ap2 results in reduced revascularization in the OIR model.

**Figure 6:**
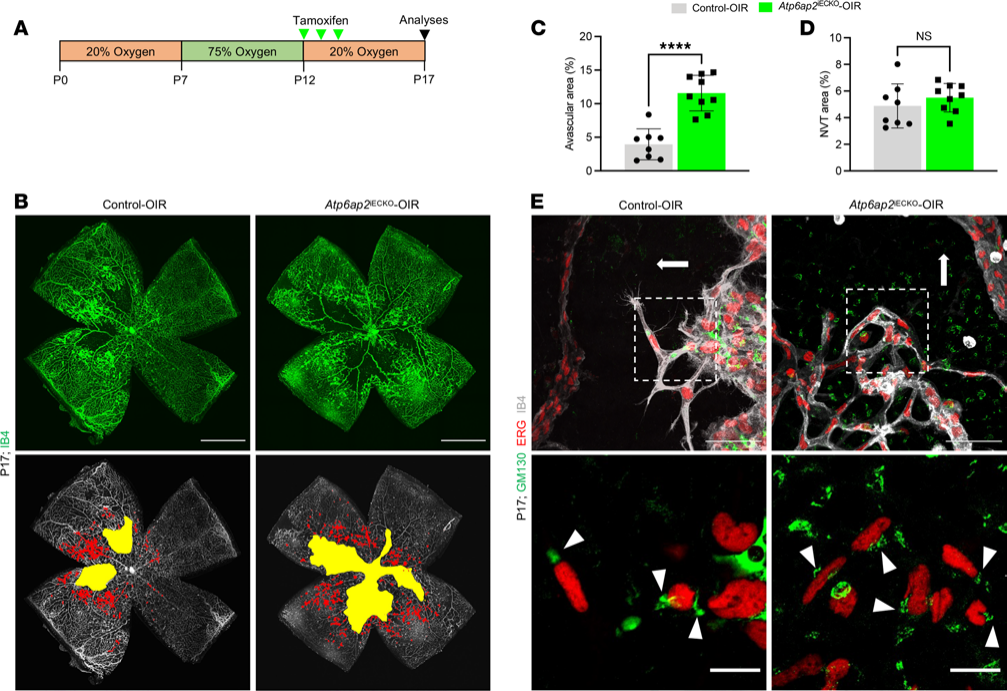
ATP6AP2 is required for proper revascularization during pathological angiogenesis in the OIR mouse model. (**A**) Schematic showing the timeline of the OIR protocol with hyperoxia phase from P7 to P12, tamoxifen administration from P12 to P14, and retina analysis at P17. (**B**) Whole-mount IB4 stained retinas showing the avascular area (yellow) and neovascular tuft (NVT) area (red) in control OIR and *Atp6ap2*^iECKO^ OIR mice at P17. Scale bars: 1,000 µm. (**C** and **D**) Quantification of the avascular area and NVT area in control OIR (*n* = 8) and *Atp6ap2*^iECKO^ OIR mice (*n* = 9) at P17. (**E**) Images of IB4^+^ vessels, ERG^+^ nuclei of ECs, and GM130^+^ Golgi apparatuses at the neovascularization edge in control OIR and *Atp6ap2*^iECKO^ OIR P17 retinas. Scale bars: 50 µm. The respective insets (white dashed-line boxes) show magnified views of tip cells. White arrowheads indicate the Golgi apparatus position in respect to the nucleus of tip ECs. White arrows indicate direction of revascularization toward the avascular area. Data are shown as mean ± SD; 2-tailed unpaired *t* test. ****P < 0.0001.

Previous works have shown *Atp6ap2* expression in fibrovascular tissues of murine and human eyes (32, 59) and indicated that ATP6AP2 is linked to patients with proliferative diabetic retinopathy (60). To expand upon these studies, we examined whether endothelial ATP6AP2 has a role in pathological angiogenesis in mice by utilizing the OIR model, which mimics conditions associated with ocular retinopathies. In these studies, *Atp6ap2* gene deletion was induced at the beginning of the hypoxic phase to assess its role during the neovascularization process (Figure 6A). Analysis of IB4-stained whole-mount retinas at P17 showed significantly impaired revascularization in *Atp6ap2*^iECKO^ OIR mice compared with control OIR mice (Figure 6, B and C). Unlike *Atp6ap2* control OIR retinas, which displayed small avascular areas and a highly recovered vasculature, *Atp6ap2* mutants exhibited large avascular areas within their retinas. Interestingly, quantification of the neovascular tuft (NVT) areas revealed no differences in tuft formation upon loss of ATP6AP2 (Figure 6D). In addition, we analyzed EC polarization during the revascularization process in the OIR mice. In control OIR mice, the Golgi apparatuses were generally polarized in front of the nuclei toward the avascular area, similar to P7 control tip cells and control siRNA–treated TeloHAECs in the scratch assay (Figure 3, E–G). Conversely, Golgi polarization in ECs was disturbed and disorganized in *Atp6ap2*^iECKO^ OIR mice (Figure 6E), as observed in ATP6AP2-deficient ECs at the leading edge of the scratch and retina vasculature (Figure 3, E–G). These results indicate that ATP6AP2 is required for establishing EC polarity during the vascular regrowth process in the OIR model.

## Discussion

ATP6AP2 directs a diverse set of physiological activities in various tissues and cell types. However, the functional role of ATP6AP2 in the endothelium is unclear. In this study, we used endothelial-specific *Atp6ap2*-KO mice to extensively characterize the role of ATP6AP2 in both physiological and pathological angiogenesis. Notably, our findings show that inactivation of *Atp6ap2* in ECs impaired postnatal retinal angiogenesis. Specifically, early deletion (P1–P3) of *Atp6ap2* led to decreased vascular outgrowth in the superficial plexus, while later ablation (P5–P7) disrupted vascularization of the deeper plexus. In addition, transcriptional profiling of ATP6AP2-deficient ECs displayed a downregulation of genes associated with various processes involved in promoting angiogenesis. Furthermore, the importance of *Atp6ap2* during pathological angiogenesis was demonstrated by the impact that loss of ATP6AP2 in ECs had on intraretinal vascularization in the OIR model. These results indicate a previously unknown and critical requirement of ATP6AP2 during in vivo angiogenesis and further support an endothelial role for ATP6AP2 function in pathophysiological settings.

A key finding in this study was the involvement of ATP6AP2 in regulating tip cell migration and polarity during angiogenic growth. *Atp6ap2*^iECKO^ mice displayed an overall reduced number of sprouts at P7, and the majority of cells at the vascular front displayed spherical nuclei typically associated with static cells. These phenotypes are consistent with impaired tip cell migration. Additionally, *Atp6ap2* mutants exhibited defective tip cell polarity. Previously, Hirose et al. demonstrated that ATP6AP2 plays a role in neuronal polarization (61), while additional studies described a conserved role for the *Drosophila* ATP6AP2 equivalent in regulating planar cell polarity (48). Thus, the roles described here for ATP6AP2 in establishing tip EC polarity and regulating polarity-associated gene expression could be shared among different cell types and among different species. We observed downregulation of both PAR3 and PARVA in *Atp6ap2*^iECKO^ mice. PAR3 and PARVA are adapter proteins important in establishing and maintaining EC polarity (50, 54). Interactions between ATP6AP2 and PAR3 in retinal homogenates and yeast 2-hybrid assays have been reported previously (62); however, to our knowledge, this is the first report of a potential mechanism involving ATP6AP2 and PARVA in the regulation of EC polarity. A disruption of these suspected interactions in ECs when *Atp6ap2* is lost may be a primary cause of the observed polarity defects; therefore, future studies aimed at understanding this mechanism are warranted. Ultimately, the aberrant EC polarity observed in *Atp6ap2*^iECKO^ mice is likely responsible, at least in part, for the significantly reduced radial vessel outgrowth, increased vascular density at the growing front, and overall altered vascular architecture. Additionally, it has been shown that, in the mouse kidney, conditional deletion of *Atp6ap2* in Foxd1^+^ stromal cells, which have been indicated as key progenitors in renal arteriole development (63), is critical for proper formation of the renal artery tree (64). Thus, ATP6AP2, acting as a functional v-ATPase subunit, contributes to the morphogenetic regulation of the renal arterial network in a non–EC-autonomous manner. Accordingly, additional roles of ATP6AP2 in various cell types that affect blood vessel formation should be taken into consideration. Moreover, considering the many reported functions of ATP6AP2, additional processes, such as actin cytoskeletal and microtubule dynamics, energy metabolism, vesicular acidification, or protein degradation (24, 61, 65, 66), could also factor into these phenotypes and remain potential areas for further investigation.

Our studies uncovered a connection between ATP6AP2 and the ECM of the endothelium. RNA-Seq analyses reveal downregulation of ECM genes in response to loss of ATP6AP2 in ECs. Mechanistically, we showed that decreases in ECM protein levels, specifically COLIII and laminin, were potentially regulated by ATP6AP2 via the ERK1/2 pathway, similar to studies in mesangial cells (58). Additional studies are needed to ascertain whether changes in EC expression of ECM components and ERK1/2 phosphorylation are directly related or happening in parallel; nonetheless, collagen and laminin are each implicated in the regulation of different phases in the angiogenesis process. For instance, exposure of microvascular ECs to COLI leads to the formation of precapillary cords, whereas these same cells do not undergo a morphology change when exposed to laminin I (16, 67). Furthermore, it is proposed that collagen is important for EC invasion and lumen formation during angiogenesis, while laminin is crucial during the vessel maturation and stabilization phase. Interestingly, and relevant to our studies, recent works have demonstrated the importance of ECM during the sprouting angiogenesis process (68–70). Thus, the direct effect of ECM proteins on EC function during angiogenesis, neovascularization, and blood vessel maturation highlights the need for investigation into their regulation. In fact, we expect that loss of ECM in the endothelium contributes to the various phenotypes observed in *Atp6ap2* mutant mice, but the mechanism and extent of their contribution are currently unknown. Overall, changes in the ECM and EC polarity in our model were not entirely surprising, as ATP6AP2 has been implicated in ECM and cell polarity regulation in multiple cell types (62, 71). Accordingly, our findings suggest that ATP6AP2 may possess similar and/or conserved roles among various cell types, and these roles could provide functional context for studying different biological processes and diseases.

Although not fully investigated in these studies, our RNA-Seq data reveal a number of critical angiogenic genes that were significantly downregulated in ATP6AP2-deficient ECs (Figure 4E). For example, the vascular ligand angiopoietin-1 (*Angpt1*) and its corresponding receptor tyrosine kinase Tek (also known as Tie2) are crucial for multiple vascular processes, including sprouting angiogenesis and EC migration (72), which were investigated in this study. Moreover, Angpt1 and Tek are important in EC permeability, quiescence, and maturation (72). From a pathological standpoint, mutations in Tek lead to venous malformations in humans (73), and angiopoietin-Tek signaling has a significant role in tumor angiogenesis (72). As another example, Notch3, one of 4 Notch receptors involved in the Notch signaling pathway, is important for vascular smooth muscle cell (VSMC) development and function, which has incidentally also been shown to be influenced by the prorenin-ATP6AP2 signaling pathway (74). Postnatal Notch3-null mutants form structurally defective smooth muscle cell layers around arterial vessels due to deficiencies in VSMC maturation and arterial differentiation (75). Furthermore, adult Notch3^–/–^ mice exhibit VSMC degeneration in the retina and brain, causing hemorrhage, loss of vessel integrity, and loss of blood-brain-barrier function (76). Interestingly, platelet-derived growth factor receptor-β (PDGFRβ), another transcript significantly decreased in our *Atp6ap2* mutant ECs (Figure 4E), is intimately involved in VSMC biology and its expression is regulated by Notch3 signaling (77). Notch3 also regulates vascular tone and flow-mediated dilation of arterial vessels, and adult Notch3 mutant mice show an increased incidence of developing heart failure under angiotensin II–induced hypertensive conditions (76). Therefore, based on the findings in our transcriptional profiling studies and the current literature, it can be speculated that endothelial ATP6AP2 plays various roles in major health-related vascular processes, such as vessel regeneration/remodeling, tumor angiogenesis, vascular malformation, hypertension, and diabetes mellitus. Understanding the cellular and molecular mechanisms involved in the role of ATP6AP2 in angiogenesis is the first step in the advancement of these medically relevant fields.

Prorenin, the natural ligand for ATP6AP2, is elevated in the plasma of patients with microvascular complications related to diabetic retinopathy (32, 78). Although systemic prorenin levels are typically lower than the pharmacological threshold for (P)RR activation, this scenario might differ during high-glucose conditions (79). For example, during high-glucose conditions, as seen in diabetic patients, there are trafficking alterations of ATP6AP2, further increasing the physical interaction between (P)RR and prorenin, which leads to upregulation of downstream fibrotic factors (80). This process is clinically relevant due to the potential therapeutic effects of targeting this receptor with an inhibitor. Blocking ATP6AP2 activity prevented cardiac fibrosis and diabetic retinopathy in hypertensive and diabetic rats, respectively (21, 81, 82). However, inhibition of ATP6AP2 during pregnancy and/or later on in life may not be an ideal treatment option clinically, as its roles in many physiological processes are not completely understood. Our study elucidated the potential role of ATP6AP2 in vascular angiogenesis and showed that loss of *Atp6ap2* had an overall detrimental effect on the growth, organization, and function of the endothelium with serious pathological events. Therefore, systemic inhibition of ATP6AP2 may have unanticipated consequences, since blood vessels are pervasive in nearly all tissues. In future studies, researchers targeting ATP6AP2 for the treatment of *Atp6ap2-*related disorders will need to consider disease settings and targeted tissue specificity of ATP6AP2.

In conclusion, this study defines the role of endothelial *Atp6ap2* during postnatal retinal angiogenesis and its involvement during neovascularization in the OIR model. Importantly, we identified ATP6AP2 as a regulator of EC polarity during physiological and pathological angiogenesis. Additionally, we found that loss of *Atp6ap2* leads to downregulation of ECM proteins, potentially due to impaired signaling in the ERK1/2 pathway in ECs. These findings provide the first evidence to our knowledge of an association between EC-type specific ATP6AP2 and the morphogenetic, well-characterized angiogenesis process that establishes the retinal vascular network.

## Methods

### Mice and treatment.

*Atp6ap2*^fl/Y^ mice were crossed with 2 EC-specific Cre lines: *Tie2-cre* for embryonic studies and *Cdh5-Cre^ERT2^* for postnatal studies (39, 42). Transgenic lines were maintained on a mixed genetic background (CD1 and C57BL/6). For embryonic studies, timed mating was carried out, designating E0.5 as noon on the day a vaginal plug was observed. To induce Cre recombination postnatally, newborn offspring were administered tamoxifen (MilliporeSigma, T5648) orally at a concentration of 100 μg on P1–P3 or P5–P7, and the retinas were analyzed at P7 or P12, respectively. Both male and female neonates were analyzed in our studies. To evaluate Cre-toxicity, *Cdh5-Cre^ERT2^* mice were crossed on mixed genetic backgrounds. Newborn pups were administered 100 μg of tamoxifen from P1 to P3, and retinas were analyzed at P7.

### Immunofluorescence analysis of mouse retina.

Whole-mount staining of retinas was performed as previously described (83). Briefly, eyeballs were removed and fixed in 4% PFA in PBS for 1 hour at 4°C. Eyeballs were washed 3 times in 1× PBS, and retinas were dissected, permeabilized in 1% TritonX-100/PBS (PBST) at room temperature for 30 minutes, and blocked with CAS-block (Thermo Fisher Scientific, 88120) at room temperature for 30 minutes. Primary antibodies were diluted in CAS-block and incubated at 4°C overnight, followed by incubation in appropriate secondary antibodies for 4 hours at room temperature. Retinas were washed in 3 times in 1× PBS and mounted on a slide using the ProLong Diamond Antifade Mountant (Thermo Fisher Scientific, P36961). The antibodies used were antibodies to IB4 (Thermo Fisher Scientific, I21411, 1:250), RFP (Abcam, ab62341), ERG1/2/3 (Abcam, ab196149, 1:200), GM130 (BD Biosciences, 610822, 1:100), Ki67 (Cell Signaling Technology, 9449, 1:100), COLIV (MilliporeSigma, AB756P, 1:100), FN (Abcam, Ab2413, 1:100), and cCASP-3 (Cell Signaling Technology, 9661, 1:100).

### Morphological analysis of retinal vasculature.

Retinal vasculature analysis was performed using ImageJ (NIH) and Angiotool analysis software (84, 85). Vascular outgrowth was measured as the distance from the optical nerve to the periphery in each leaflet of the retina and averaged. Vascular density and the number of branching points were quantified using whole-retinal images in the Angiotool analysis software. The numbers of sprouts and filopodia were counted manually at the vascular front in 200× and 1,000× images, respectively. Quantification of ERG^+^ nuclei of ECs was performed at the vascular front of 600× image using ImageJ. Nuclear ellipticity was determined by dividing the measured nuclear height by nuclear width of 10–12 ERG^+^ cells per image at the vascular front and presented as a ratio. For cell proliferation analysis, ERG^+^/Ki67^+^ cells were quantified at the vascular front of 400× image using ImageJ.

### Isolation of murine lung ECs.

Isolation of murine lung ECs was performed as previously described (86). Briefly, lungs were harvested at P7, minced, and digested in collagenase I (Thermo Fisher Scientific, 17-100-17)/dispase (Corning, 354235) buffer at 37·C for 30 minutes under constant rotation. The digested tissue was triturated 10 times through an 18G needle to dissociate the clumps and filtered using 70 µm nylon mesh (VWR, 10199-656) to remove any cell debris. The cell suspension was centrifuged at 250*g* at 4°C for 5 minutes. Single-cell suspensions were incubated with sheep anti–rat IgG dynabeads (Invitrogen, 11035) coated with CD31 antibody (BD Pharmingen, 553370) at room temperature for 20 minutes with agitation. Lastly, CD31^+^ cells were isolated using a magnetic rack separator, and either RNA or protein was isolated from the cells for downstream analysis. Purity of iLECs was determined and confirmed via immunoblot analysis (Supplemental Figure 11).

### Isolation of murine retinal ECs.

Retinal ECs were isolated from 6 to 8 pooled retinas of control and *Atp6ap2*^iECKO^ mice at P7 for each sample. Isolation was performed using the Neural Tissue Dissociation Kit (P) (Miltenyi Biotec, 130-092-628) with minor modifications. Briefly, retinas were dissected in cold HBBS buffer supplemented with FBS, penicillin-streptomycin (Thermo Fisher Scientific, 15070063), and HEPES (Thermo Fisher Scientific, 15630080). Retinas were digested in Enzyme mix 1 (Enzyme P and buffer X) at 37°C for 15 minutes under constant rotation. Following the digestion, Enzyme mix 2 (enzyme A and buffer Y; Miltenyi Biotec) was added to the sample and incubated in a 37°C water bath for 10 minutes. The sample was mixed by pipetting every 3 minutes during the incubation. The cell suspension was centrifuged at 800*g* at 4°C for 5 minutes. The cells were then filtered using a 70 µm cell strainer. Cells were centrifuged again (800*g*, 4°C, 5 minutes) and resuspended in buffer containing dynabeads (Invitrogen, 11035) coated with CD31 antibody (BD Pharmingen, 553370) at 4°C for 30 minutes under constant rotation. Lastly, CD31^+^ cells were isolated using a magnetic rack separator and RNA was isolated from the cells for analysis.

### Western blot.

Protein was isolated from the cells using the RIPA lysis buffer (Thermo Fisher Scientific, 89901) supplemented with protease inhibitor cocktail (Thermo Fisher Scientific, 78430) and phosSTOP (MilliporeSigma, 04906845001). Cell lysates were centrifuged at 13,000*g* at 4°C for 10 minutes, and supernatants were collected. Protein concentrations were determined using the Pierce Coomassie (Bradford) protein assay kit (Thermo Fisher Scientific, 23200). Laemmli buffer (Bio-Rad, 1610737) supplemented with 2-mecrapto-ethanol (Bio-Rad, 1610710) was added to the protein samples and boiled at 95°C for 5 minutes for denaturation. Each protein lysate was separated on 4%–20% Mini-PROTEAN TGX Precast gels (Bio-Rad, 4568094) and blotted onto 0.2 μm PVDF membrane (Bio-Rad, 1704156). Membranes were blocked for 30 minutes with 5% BSA in TBST (0.1% Tween 20 in TBS) and incubated in primary antibodies diluted in the blocking buffer at 4°C overnight. Following primary antibody incubation, membranes were washed in TBST and incubated with appropriate secondary antibodies for 1 hour at room temperature. Target proteins were detected using the LI-COR Odyssey imaging system. Band densitometry was quantified using the ImageJ software. See complete unedited blots in the supplemental material.

The following primary antibodies were used for the Western blot analysis: anti–β-actin (Cell Signaling Technology, 3700S, 1:5,000), anti-ATP6AP2 (MilliporeSigma, HPA003156, 1:1,000), anti–p44/42 MAPK (ERK1/2) (Cell Signaling Technology, 4695, 1:1,000), anti–p-p44/42 MAPK (pERK1/2) (Cell Signaling Technology, 9101, 1:1,000), anti-COLIII (Abcam, ab184993, 1:1,000), anti-laminin (MilliporeSigma, L9393, 1:1,000), anti-partitioning-defective 3 (Par3) (MilliporeSigma, 07-330, 1:1,000), anti-PARVA (Cell Signaling Technology, 8190, 1:1,000), anti–VE-cadherin (Cell Signaling Technology, 2500s, 1:1,000), anti-αSMA (Abcam, Ab5694, 1:2,500), and anti-desmin (Abcam, Ab32362, 1:1,000).

### qPCR.

Total RNA was extracted from the samples using the GeneJET RNA Purification Kit (Thermo Fisher Scientific, K0732) following the manufacturer instructions. To determine the mRNA expression levels, 1 μg of extracted RNA was transcribed into cDNA using the iScript Reverse Transcription Supermix kit (Bio-Rad, 1708840). qPCR was performed using the PerfeCTa SYBR Green SuperMix (Quantabio, 95071) on CFX96 system (Bio-Rad) following manufacturer instructions. The list of qPCR primers used in this study is included in Supplemental Table 1. Relative gene expression was determined using the ΔΔCt method. Three independent biological replicates were used, and 3 technical replicates were performed per sample.

### Cell culture and stimulation.

TeloHAECs (ATCC, CRL-4052) were cultured in EBM-2 basal media (Lonza, 3156) supplemented with EGM-2 Bullet kit (Lonza, 3162) at 37°C and 5% CO_2_. For stimulation studies, cells transfected with *Atp6ap2* siRNA or control siRNA were serum starved overnight and stimulated with 20 nM prorenin (Cayman Chemical, 10007599) for indicated time points. Following stimulation, protein was extracted from each treatment for analysis via immunoblotting.

### RNA interference.

TeloHAECs were transfected with a pool of *Atp6ap2* (Dharmacon, L-013647-01-0005) and nontargeting control siRNAs (Dharmacon, D-001810-10-05) using Lipofectamine 3000 (Thermo Fisher Scientific, L3000015) following manufacturer instructions. The final concentration of siRNA solution was 200 nM. Cells were harvested and analyzed for knockdown efficiency 48 hours after transfection.

### Scratch wound healing assay.

Scratch assays were performed on confluent control and *Atp6ap2* siRNA–transfected TeloHAECs. A horizontal and vertical scratch was created in each well using a 200 µL pipet tip followed by 2 washes with EGM-2 media. Phase contrast images were taken right after the scratch (0 hour) and 40 hours afterward. Percentage of wound closure and rate of migration was analyzed and determined using the ImageJ software.

### Cell staining and polarity analyses.

TeloHAECs were cultured on gelatin-coated coverslips. Cells were stained 12 hours following the scratch for polarity studies. For staining, cells were fixed in 4% PFA for 10 minutes, permeabilized in 0.1% NP40/PBS for 15 minutes, and blocked in CAS-block for 30 minutes at room temperature. Cell-coated coverslips were incubated in primary antibodies diluted in CAS-block for 1 hour at room temperature, secondary antibodies, and DAPI diluted in CAS-block for 1 hour at room temperature. After several washes in 1× PBS, coverslips were air-dried and mounted on a slide using the ProLong Diamond Antifade Mountant (Thermo Fisher Scientific). After mounting, random fields of view for each treatment were imaged with a Nikon confocal microscope (total original magnification, 400×). Cell polarity toward the scratch was determined by measuring the nucleus/Golgi apparatus axis angle in 10–12 cells per image from 3 independent experiments using ImageJ software (NIH). Antibodies used for cell immunofluorescence analysis were: DAPI (Invitrogen, R37606, 1:500), Phalloidin (F-Actin) (Invitrogen, A12379, 1:200), and GM130 (Cell Signaling Technology, 12480, 1:100).

### Three-dimensional fibrin gel bead sprouting assay.

The 3-dimensional in vitro bead assay was performed and analyzed as previously described (87). Briefly, siRNA transfected TeloHAECs were coated onto Cytodex 3 beads (Cytivia, 17048501) at a concentration of 400 cells per bead in 1 mL of EGM2 media incubated for 4 hours at 37°C with gentle shaking every 20 minutes, transferred to a 25 cm^2^ flask, and incubated overnight in 7 mL of EGM2 media at 37°C and 5% CO_2_. The next day, TeloHAEC-coated beads were resuspended in 2.5 mg/mL fibrinogen (MilliporeSigma, F8630) in PBS. Aprotinin (MilliporeSigma, A1153) was added to the fibrinogen/bead solution at a concentration of 4 U/mL. Thrombin (MilliporeSigma, 605157) was added to the center of a well at a concentration of 50 U/mL followed by the fibrinogen/bead solution in a glass-bottom 24-well plate. The gels were allowed to solidify for 5 minutes at room temperature before it was placed at 37°C and 5% CO_2_ for 30 minutes. Normal human lung fibroblasts (Lonza, 2512) were plated on top of the fibrin gel at a concentration of 10,000 cells per well. Media was changed every other day, and sprouts were analyzed 3–5 days following sprout formation.

### RNA-Seq and gene expression analysis.

Total RNA was extracted from iLECs and quantified using Qubit RNA High Sensitivity Assay Kit (Thermo Fisher Scientific, Q32852). RNA integrity was determined using the Bioanalyzer RNA 6000 Nano assay kit (Agilent, 5067-1511). RNA library construction was performed with the TruSeq RNA Library Prep Kit v2 (Illumina, RS-122-2001) according to the manufacturer instructions. The resulting mRNA library was quantified using Qubit dsDNA High Sensitivity Assay Kit (Thermo Fisher Scientific, Q32851) and verified using the Bioanalyzer DNA1000 assay kit (Agilent, 5067-1505). Verified samples were sequenced using the NextSeq 500/550 High Output Kit v2.5 (150 Cycles) (Illumina, 20024907) on a Nextseq 550 system (Illumina, SY-415-1002). Sequenced reads were aligned to the mouse (mm10) reference genome with RNA-Seq alignment tool (version 2.0.1). The aligned reads were used to quantify mRNA expression and determine differentially expressed genes using the RNA-Seq Differential Expression tool (version 1.0.1). Both alignment and differential expression analysis were performed using the tools in the BaseSpace Sequence Hub. Overrepresentation analysis of differentially expressed genes was performed using the WEB-based Gene SeT AnaLysis Toolkit (WebGestalt) (88). Sequencing data have been deposited in the Gene Expression Omnibus (GEO) database with accession no. GSE179431.

### OIR studies.

To induce retinal pathological angiogenesis, the OIR model was used, as previously described (89). From P7 to P12, pups were placed in a hyperoxia chamber (Biospherix, ProOx110) with 75% oxygen; at P12, pups were returned to room air until P17. Tamoxifen was administered to pups at a concentration of 200 μg from P12 to P14 to induce Cre recombination. Retinas were collected at P17 and processed as outlined in the section of immunofluorescence analysis of mouse retina. Quantification of the avascular and NVT area was performed using an automated OIR retinal image analysis software (90).

### Statistics.

Data analysis was performed using GraphPad Prism version 9.0.0 for MacOS, GraphPad Software (www.graphpad.com). Quantified data are presented as bar graphs of mean ± SD. Unpaired 2-tailed Student’s *t* tests assuming equal variance were used to determine statistical significance between 2 groups with *P* < 0.05 considered statistically significant.

### Study approval.

All animal experiments were performed in accordance with Tulane University’s IACUC policy.

## Author contributions

NRP and SMM developed and designed the experiments. NRP, RKC, AB, and YL performed the experiments. NRP, MCP, and SMM wrote the manuscript.

## Supplementary Material

Supplemental data

## Version 1 08/23/2022

In-Press Preview

## Version 2 10/10/2022

Electronic publication
