# Supplementary material for: Endothelial cell polarity and extracellular matrix composition require functional ATP6AP2 during developmental and pathological angiogenesis
Source: JCI Insight. 2022 Oct 10;7(19):e154379. doi: 10.1172/jci.insight.154379 (PMC9675464; doi:10.1172/jci.insight.154379)
Supplement: Supplemental data [file jciinsight-7-154379-s118.pdf]

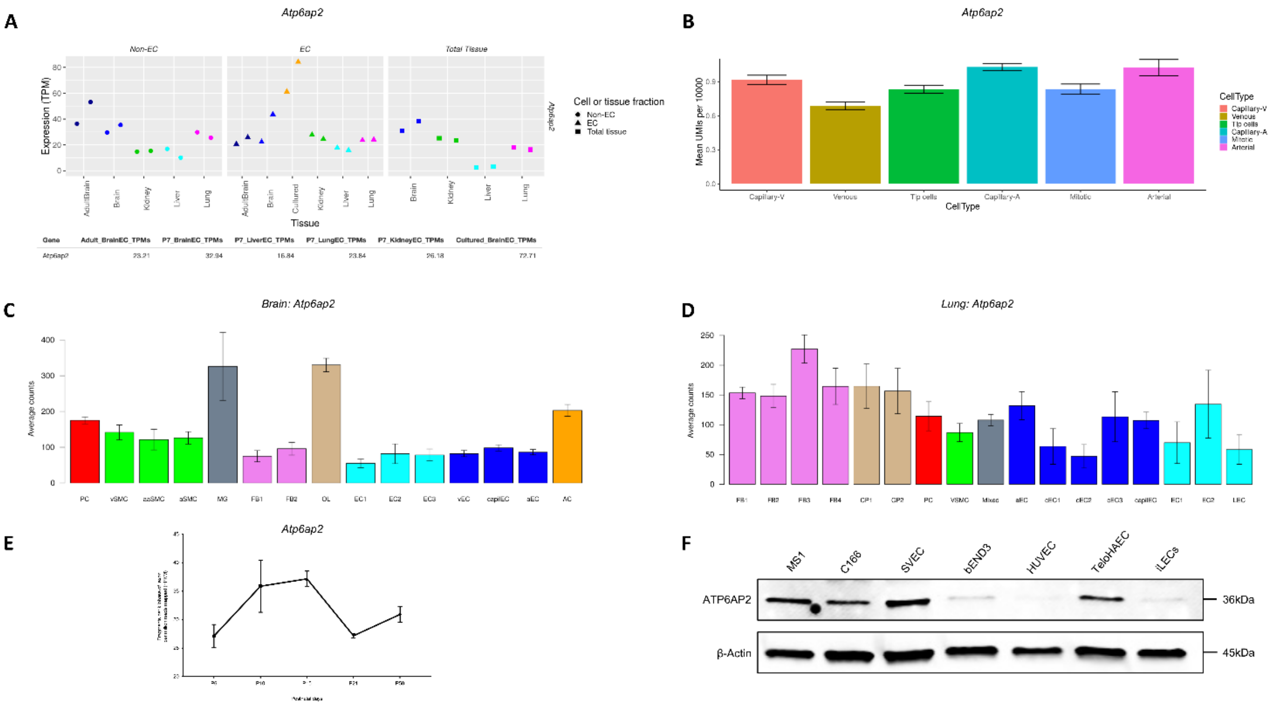

Supplementary Figure 1. ATP6AP2 is robustly expressed in vascular endothelium. A) Analysis of *Atp6ap2* expression levels in non-EC, ECs and total tissue of P7 brain, kidney, liver, lung, and adult brain using the Vascular Endothelial Cell Trans-omics Resource Database (VECTRDB). **B**) Analysis of *Atp6ap2* expression levels in P7 isolated brain ECs and its subtypes using single cell RNA-seq data in VECTRDB. **C, D**) *Atp6ap2* expression levels in adult murine brain and its support cells (**C**) and lung and its support cells (**D**), as determined using the single cell RNA-seq data from the following database: http://betsholtzlab.org/VascularSingleCells/database.html. Brain data: PC - Pericytes; SMC - Smooth muscle cells; MG - Microglia; FB - Vascular fibroblast-like cells; OL - Oligodendrocytes; EC - Endothelial cells; AC - Astrocytes; v - venous; capil- capillary; a - arterial; aa - arteriolar; 1,2,3- subtypes. Lung data: FB - Vascular fibroblast-like cells; CP - Cartilage perichondrium; PC - Pericytes; VSMC - Vascular smooth muscle cells; EC - Endothelial cells; capil - capillary; a - arterial; c - continuum; L - Lymphatic; 1,2,3,4 – subtypes. **E**) Expression levels of *Atp6ap2* in murine retinal endothelial cells during postnatal development determined using the available bulk RNA sequencing data[38]. **F**) Western blot analysis of ATP6AP2 and β-ACTIN expression in various murine and human primary and transformed ECs.

A

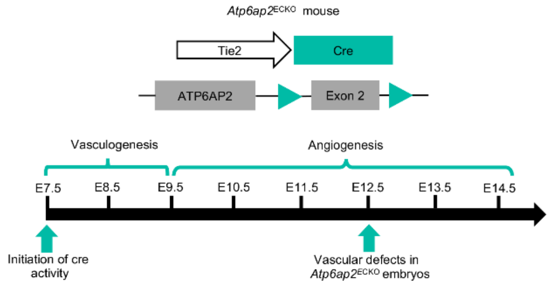

B Number of P21 mice obtained from *Tie2*-Cre X *Atp6ap2* X^{fl}/X^{fl} matings:

Genotype	*Atp6ap2* X^{fl}/Y	*Atp6ap2* X^{fl}/Y;*Tie2*-Cre	*Atp6ap2* X^{fl}/X^{WT}	*Atp6ap2* X^{fl}/X^{WT};*Tie2*-Cre
Observed mice	23 (29%)	0 (0%)	33 (42%)	23 (29%)
Expected mice	19.75 (25%)	19.75 (25%)	19.75 (25%)	19.75 (25%)

C

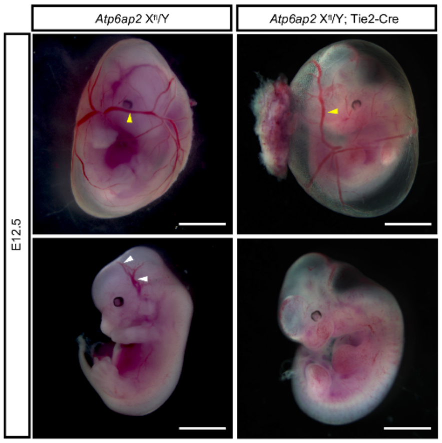

Supplementary Figure 2. Endothelial-specific deletion of *Atp6pa2* induces embryonic lethality. A) Strategy for EC-specific deletion of *Atp6ap2* during embryogenesis using the *Tie2*-Cre driver line and summary of the timepoint when vascular defects were observed. **B**) Genotype frequencies of postnatal day 21 pups from *Tie2*-Cre and *Atp6ap2* X^{fl}/X^{fl}mating pairs. No postnatal deaths were observed in pups that were born. **C**) Whole mount, bright-field images of *Atp6ap2* X^{fl}/Y and *Atp6ap2* X^{fl}/Y;Tie2-cre yolk sacs and embryos at E12.5. Note that the large, perfused vessels within the brain of control embryos (white arrowheads) are absent in *Atp6ap2* X^{fl}/Y;Tie2-cre mice. *Atp6ap2* mutants are also underdeveloped, however yolk sac vessels (yellow arrowheads) appear relatively normal compared to *Atp6ap2* X^{fl}/Y embryos (scale bars: 2 mm).

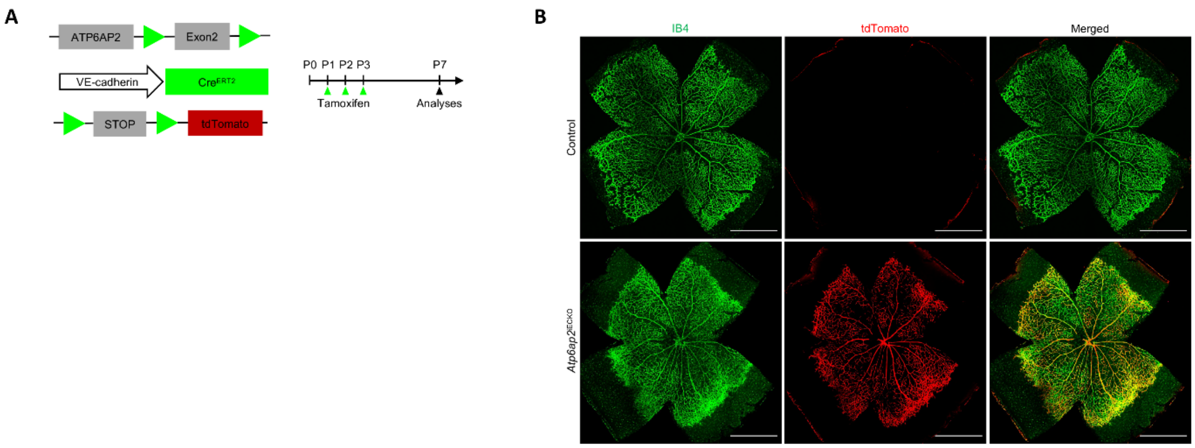

Supplementary Figure 3. Inducible EC-specific Cre recombination in *Atp6ap2^{iECKO}* mice. **A**) Schematic representation of the experimental timeline for monitoring EC-specific Cre recombination using the Rosa-tdTomato reporter integrated in *Atp6ap2^{iECKO}* mice, which harbor *VE-cadherin* (*Cdh5*) Cre-ERT2. **B**) Whole-mount images of control and *Atp6ap2^{iECKO}* P7 retinas showing IB4^{+} vessels and tdTomato expression only in the ECs of *Atp6ap2^{iECKO}* mice (scale bars: 1000 μm).

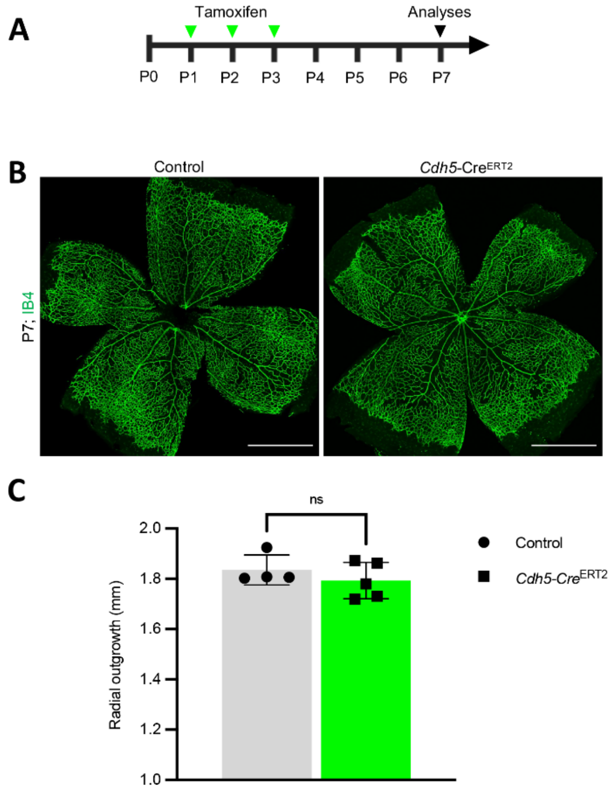

Supplementary Figure 4. Tamoxifen delivery to inducible EC-specific Cre-driver mice does not impact retinal outgrowth. A) Schematic outlining the stages of tamoxifen administration and retina analysis in neonate pups. **B)** Whole-mount images of control and *Cdh5*-CreERT2 retinas displaying IB4+ vessels (scale bars: 1000 μm). **C)** Quantification of radial outgrowth in control and *Cdh5*-CreERT2 mice at P7 (n=4).

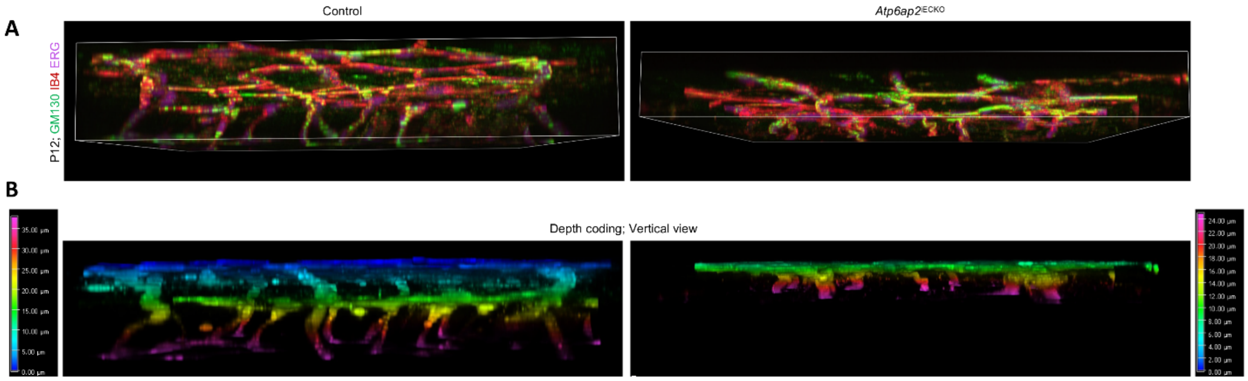

Supplementary figure 5. Endothelial *Atp6ap2* deletion results in impaired vertical sprouting during postnatal retinal angiogenesis. A) 3D vertical view images of the capillary plexus and deep layer vessel sprouts in control and A*tp6ap2*^{iECKO} retinas stained for GM130, IB4, and ERG at P12 (n=3). **B)** 3D vertical view images of the deep layer capillary plexus represented in depth coding.

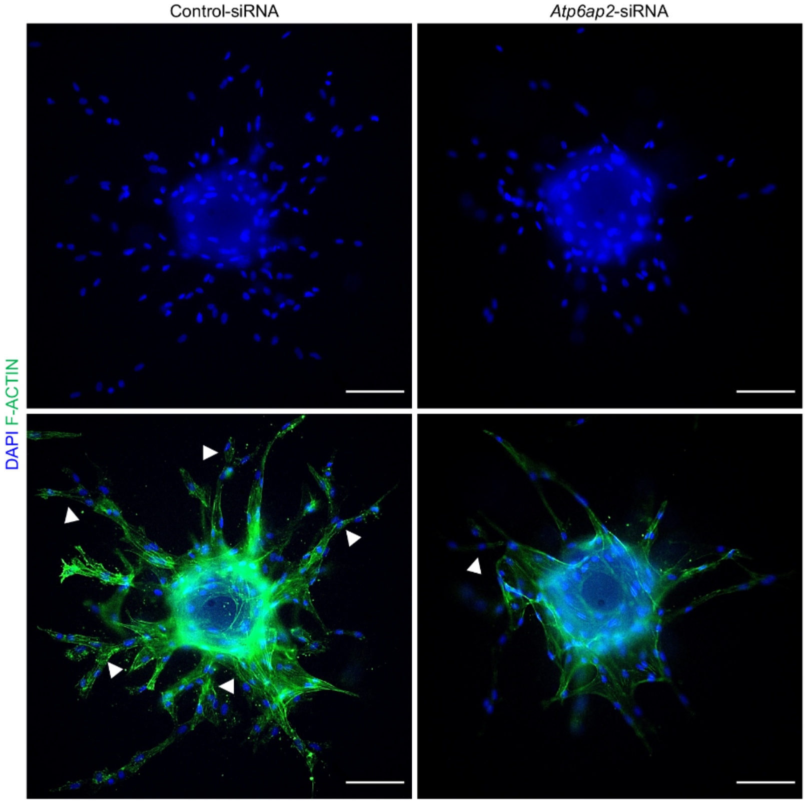

Supplementary figure 6. Loss of *Atp6ap2* in ECs results in impaired sprouting *in vitro*. Fibrin gel bead assays of control and *Atp6ap2*-siRNA treated TeloHAECs immunofluorescently labeled for DAPI and F-ACTIN. Beads in both conditions are coated with similar number of ECs. White arrowheads indicate bifurcated sprouts (scale bars: 200 μm).

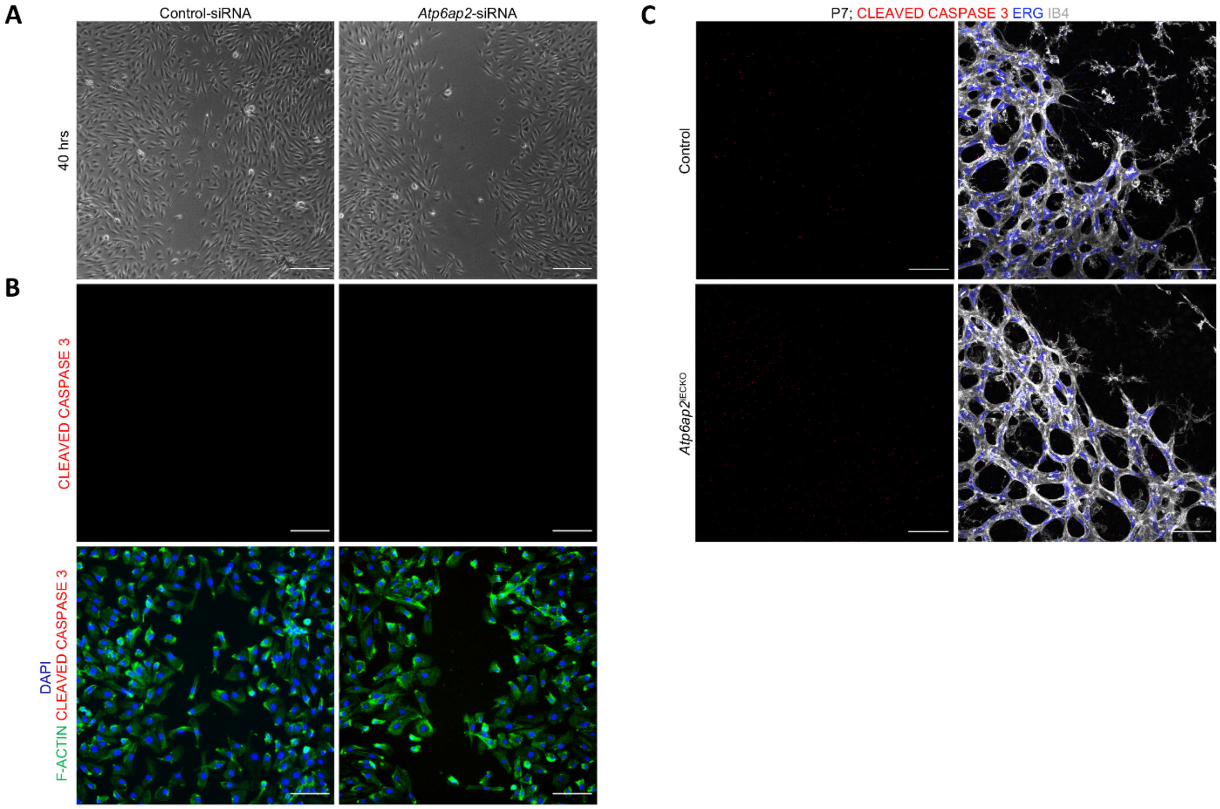

Supplementary figure 7. Apoptosis is not associated with *Atp6pa2* deficient ECs. A) Representative bright-field images of scratch assays using TeloHAECs transfected with control or *Atp6ap2*-siRNA after 40 hours (scale bars: 200 μm). **B)** Control and *Atp6ap2*-siRNA treated TeloHAECs immunolabeled for CLEAVED CASPASE 3, F-ACTIN, and DAPI in (scale bars: 200 μm). **C)** Close-up images of the vascular front in control and *Atp6ap2*^{iECKO} P7 retinas immunolabeled for CLEAVED CASPASE 3, ERG, and IB4 (scale bars: 50 μm).

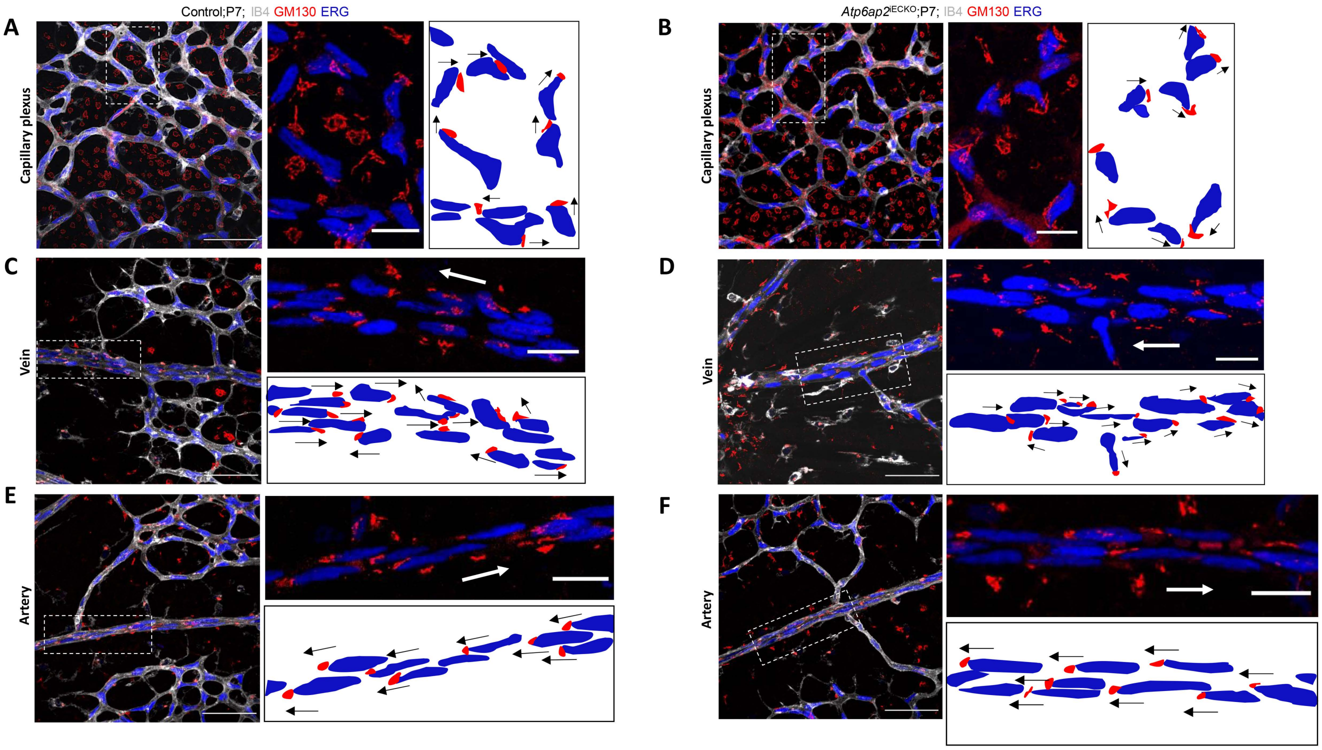

Supplementary figure 8. Endothelial cell polarity appears unaltered in capillaries, veins, and arteries of *Atp6ap2*$^{\mathbf{iECKO}}$ retinas. Representative images of control and A*tp6ap2*^{iECKO} retinas stained for GM130, ERG, and IB4 in the capillary plexus **(A, B)**, vein **(C, D),** and artery **(E, F)** at P7 (n=7; scale bars: 50 μm and 15 μm for inset). Black arrows denote the direction of EC polarization; white arrows indicate direction of blood flow in veins and arteries.

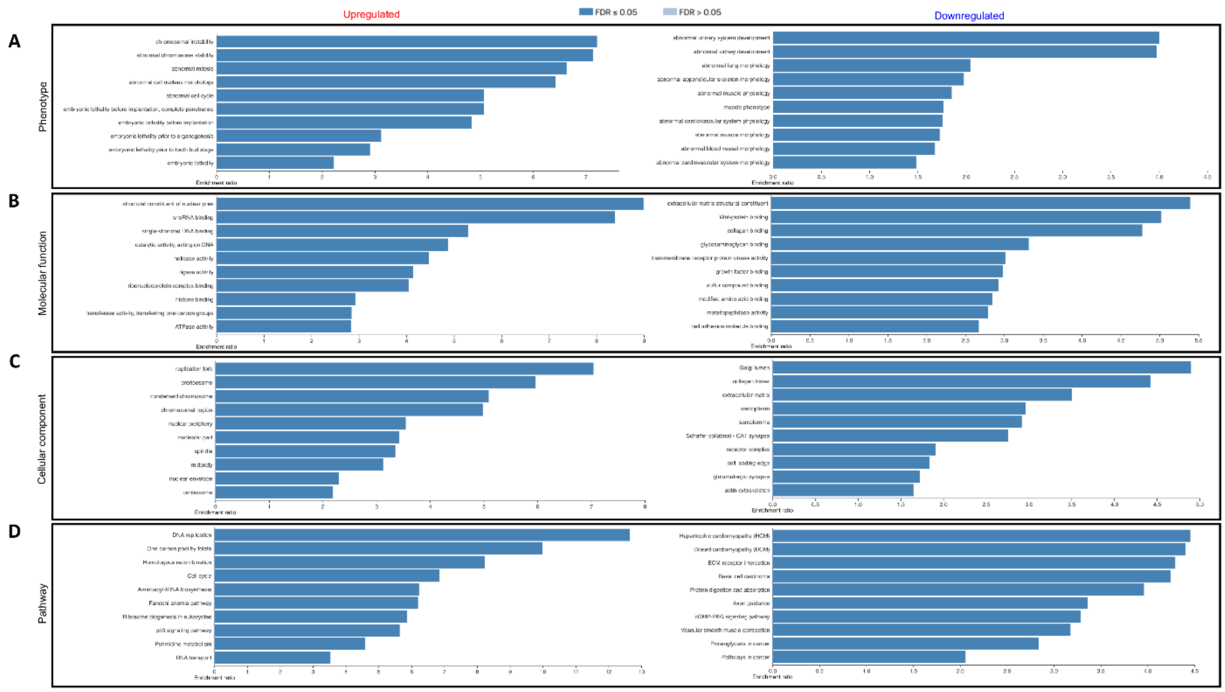

Supplementary Figure 9. Gene ontology (GO) analysis of differentially expressed genes following loss of *Atp6ap2* in ECs. A-D) Top terms enriched in upregulated and downregulated genes associated with (**A**) phenotype, (**B**) molecular function, (**C**) cellular component, and (**D**) pathway following RNA-seq analysis of *Atp6ap2*-deficient iLECs.

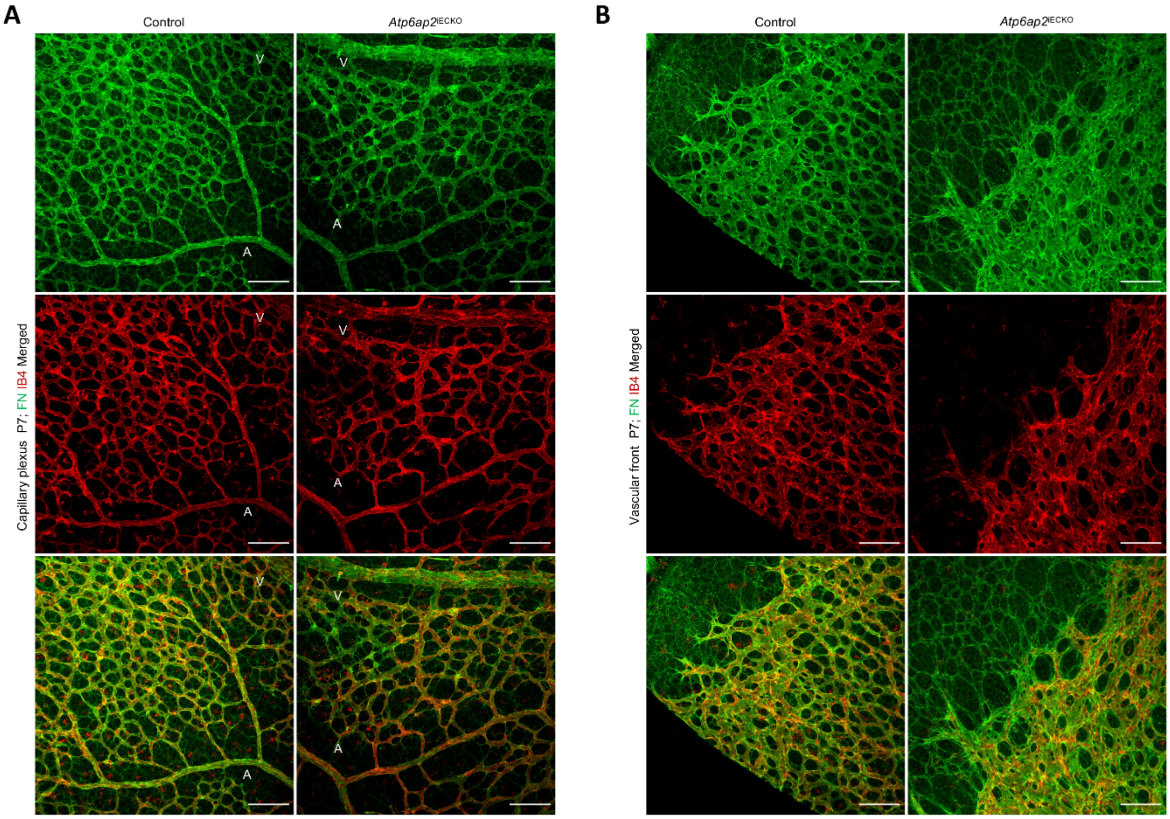

Supplementary figure 10. FIBRONECTIN expression is unaltered in the retinal vasculature of A*tp6ap2*^{iECKO} mice. Representative images of control and A*tp6ap2*^{iECKO} retinal vasculature fluorescently immunolabeled for FIBRONECTIN (FN) and IB4 in the **A)** capillary plexus and **B)** vascular front at P7 (n=4; A, artery; V, vein; scale bar: 100μm).

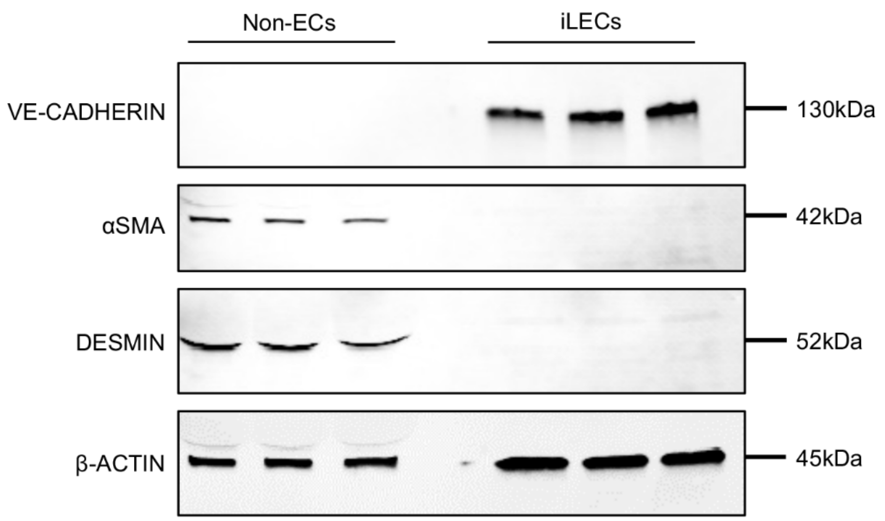

Supplementary figure 11. Expression of VE-CADHERIN, αSMA, and DESMIN in non-ECs and isolated lung ECs (iLECs) fractions. Immunoblot analysis show VE-CADHERIN was only detected in iLECs, while α-SMA and DESMIN were detected in the non-ECs fraction following EC isolation. β-ACTIN served as the protein loading control.

Supplementary Table 1: List of qPCR primer sequence

Gene	Forward (5' to 3')	Reverse (5' to 3')
β-actin (Mouse)	CTCTTTTCCAGCCTTCCTTCT	AGGTCTTTACGGATGTCAACG
GAPDH (Human)	TGCACCACCAACTGCTTAGC	GGCATGGACTGTGGTCATGAG
Atp6ap2 (Mouse)	CCAGTTTGTTGTCTCGTCATAAGC	GCGTTCCCACCATAGAGACTG
ATP6AP2 (Human)	GTGTTTTGGGGAACGAGTTTAGT	TCCTGGTATAGGCCAATTTCCA
α-parvin (Mouse)	TCCCCAAATCACCCACTCC	AGGTTGATGGCGTTCATTCCT
Col3a1 (Mouse)	CTGTAACATGGAAACTGGGGAAA	CCATAGCTGAACTGAAAACCACC
Lama2 (Mouse)	TCCCAAGCGCATCAACAGAG	CAGTACATCTCGGGTCCTTTTTC
